# Synthesis, Characterization, and Antioxidant Activity Evaluation of New N-Methyl Substituted Thiazole-Derived Polyphenolic Compounds

**DOI:** 10.3390/molecules30061345

**Published:** 2025-03-17

**Authors:** Alexandra Cătălina Cornea, Gabriel Marc, Ioana Ionuț, Cristina Moldovan, Anca Stana, Smaranda Dafina Oniga, Adrian Pîrnău, Laurian Vlase, Ilioara Oniga, Ovidiu Oniga

**Affiliations:** 1 Department of Pharmaceutical Chemistry, “Iuliu Hațieganu” University of Medicine and Pharmacy, 41 Victor Babeș Street, RO-400012 Cluj-Napoca, Romania; alexandra.cata.cornea@elearn.umfcluj.ro (A.C.C.); ionut.ioana@umfcluj.ro (I.I.); cmoldovan@umfcluj.ro (C.M.); stana.anca@umfcluj.ro (A.S.); ooniga@umfcluj.ro (O.O.); 2 Department of Organic Chemistry, “Iuliu Hațieganu” University of Medicine and Pharmacy, 41 Victor Babeș Street, RO-400012 Cluj-Napoca, Romania; 3 Department of Therapeutic Chemistry, “Iuliu Hațieganu” University of Medicine and Pharmacy, 12 Ion Creangă Street, RO-400010 Cluj-Napoca, Romania; smaranda.oniga@umfcluj.ro; 4 National Institute for Research and Development of Isotopic and Molecular Technologies, 67-103 Donath Street, RO-400293 Cluj-Napoca, Romania; adrian.pirnau@itim-cj.ro; 5 Department of Pharmaceutical Technology and Biopharmaceutics, “Iuliu Hațieganu” University of Medicine and Pharmacy, 41 Victor Babeș Street, RO-400012 Cluj-Napoca, Romania; laurian.vlase@umfcluj.ro; 6 Department of Pharmacognosy, “Iuliu Hațieganu” University of Medicine and Pharmacy, 12 Ion Creangă Street, RO-400010 Cluj-Napoca, Romania; ioniga@umfcluj.ro

**Keywords:** Hantzsch heterocyclization, polyphenols, thiazole derivatives, ROS, oxidative stress, in vitro, antioxidant, DFT

## Abstract

Reactive oxygen species play a significant role in various pathological conditions, driving the need for novel, potent antioxidants. While polyphenols are known for their antioxidant properties, their limited stability and bioavailability present challenges for therapeutic applications. To address these limitations, a series of novel thiazolyl-polyphenolic compounds was synthesized via a multi-step synthetic route incorporating Hantzsch heterocyclization in the final step. The synthesized compounds **7a**–**k** were structurally characterized using spectroscopic techniques, including NMR, MS, and IR. In silico thermodynamic calculations, including HOMO–LUMO gap and bond dissociation enthalpy (BDE) calculations, revealed a promising antioxidant profile for these compounds and indicated that the substitution in position 2 of the thiazole ring does not substantially influence the antioxidant activity conferred by the catechol moiety in position 4. The antioxidant capacity of the synthesized compounds was experimentally validated using a panel of six distinct assays: two radical scavenging assays (ABTS and DPPH) and four electron transfer-based assays (RP, TAC, FRAP, and CUPRAC). The in vitro evaluation demonstrated that compounds **7j** and **7k** exhibited significantly enhanced antioxidant activity compared to the established antioxidant standards, ascorbic acid and Trolox. These findings suggest that the strategic modifications in position 2 of the thiazole scaffold represent a promising direction for future research aimed at developing novel therapeutic agents with enhanced antioxidant properties. The present study is limited to the in vitro evaluation of compounds based on the N-methyl substituted thiazole scaffold, but future studies can include modifications such as changing the substituent on the thiazole nitrogen, the hydrazone linker or possible insertion of substituents in position 5 of thiazole ring of substituents with various electronic or physico-chemical properties.

## 1. Introduction

The concept of oxidative stress was first defined in 1985 as an imbalance between the production of reactive oxygen species (ROS) and their detoxification by endogenous antioxidant systems. These ROS include the superoxide radical O_2_^•−^, singlet oxygen ^1^O_2_, hydroxyl radicals ^•^OH, and hydrogen peroxide H_2_O_2_, which are generated as byproducts of normal metabolic processes such as protein phosphorylation and apoptosis. Within the biological systems, ROS production can also arise from alterations in intracellular redox reactions during aerobic metabolism, involving oxygen, catalyzed by oxidases such as lipoxygenases (LPO), xanthine oxidase (XO), NADPH oxidases, cyclooxygenases (COX), and cytochrome P450 enzymes. In addition to the endogenous sources, ROS generation can be attributed to various exogenous factors, including industrial chemicals, air pollution, pesticides, tobacco smoke, exposure to ionizing (X-ray) and ultraviolet (UV) radiations, viral and bacterial infections, certain pharmaceuticals, and ozone [1,2,3].

Prolonged exposure to oxidative stress has been implicated in the pathophysiology of numerous diseases, including cardiovascular, autoimmune, oncological, and neurodegenerative conditions. At a molecular level, oxidative stress can induce damage to proteins, lipids, and nucleic acids, leading to functional disruption and, notably, DNA damage. The formation of 8-oxo-2′-deoxyguanosine (8-OHdG), a well-established marker of oxidative DNA damage and mutagenesis, is a characteristic consequence of sustained oxidative stress. This DNA damage, potentially resulting from aberrant methylation of CpG islands in gene promoters, can contribute to the loss of epigenetic information [2,3,4,5,6].

Antioxidants constitute a crucial defense against oxidative stress by neutralizing the reactive oxygen species. These molecules mitigate oxidative damage through mechanisms such as hydrogen atom donation (HAT) or single electron transfer (SET), thereby inhibiting the harmful oxidative processes within biological systems. Contemporary research indicates that timely administration of antioxidants can be efficacious in preventing or attenuating the progression of diseases associated with oxidative stress by mitigating ROS-induced damage [1,7,8]. Prominent examples of antioxidants include ascorbic acid (vitamin C), retinol (vitamin A), α-tocopherol (vitamin E), flavonoids, phenolic acids, and stilbenes. Natural sources of antioxidants are abundant in fruits, vegetables, teas, algae, and cereals. Polyphenols constitute a significant class of natural antioxidants, and their biological activities and health benefits have been extensively investigated. These studies have demonstrated the capacity of polyphenols to neutralize reactive oxygen species (ROS) and suppress oxidative stress [9,10,11,12,13].

The thiazole core’s chemical versatility enables its participation in a broad spectrum of pharmacological activities such as: antioxidant, antimicrobial, anti-inflammatory, and anticancer effects [14,15,16,17,18,19]. Some thiazole derived compounds such as tiazofurin are drug candidates and many compounds such as ritonavir, sulfathiazole, abafungin, meloxicam, thiabendazole, nitazoxanide, famotidine, ravuconazole, aztreonam, or febuxostat were approved for human use [20]. Notably, thiazole derivatives have demonstrated potential in modulating oxidative stress by inhibiting key enzymes such as NADPH oxidases. Furthermore, they can suppress inflammatory mediators, suggesting therapeutic applications in conditions characterized by chronic inflammation and oxidative damage, such as rheumatoid arthritis and cancer. Importantly, the incorporation of the thiazole ring into antioxidant molecular architectures has emerged as a promising strategy for developing novel therapeutic agents targeting the interconnected pathways of oxidative stress and inflammation. This strategy leverages the thiazole ring’s capacity to contribute to antioxidant activity, often by influencing key properties like bond dissociation energies (BDEs) of nearby functional groups involved in ROS neutralization [21,22,23].

Based on literature data and encouraging preliminary results obtained by our research group [24], we designed and synthesized a novel series of thiazolyl-polyphenolic compounds. These prior findings indicated that the combination of these two pharmacophores within a single molecular entity yielded compounds exhibiting substantial antioxidant activity, comparable to established antioxidant standards. Building upon the core structure reported previously [15], we extended the series by modulating the substituent in position 2 of the thiazole ring. Specifically, we introduced a second phenolic fragment linked via a hydrazone bridge containing one, two, or three OH phenolic groups. This modification aimed to investigate the influence of this additional phenolic moiety on the antioxidant activity induced by catechol substituent in position 4 and to determine whether increasing the number of phenolic hydroxyl groups potentiates this activity.

## 2. Results

### 2.1. Chemical Synthesis of the Compounds

**Scheme 1:**
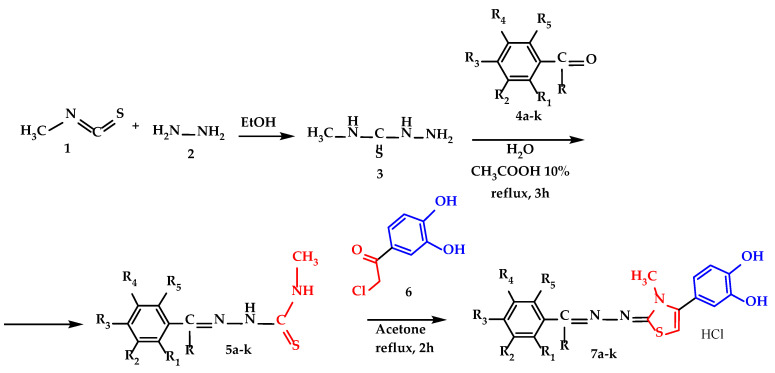
Synthetic procedure for the thiazolyl-polyphenolic derivate compounds **7a**–**k**, where R=H, except compound **7f,** where R=-CH_3_ and description of R_1_–R_5_ fragments are presented in Table 1.

All compounds were synthesized through three successive steps which were described in Scheme 1. The first step was the synthesis of 4-methyl-3thiosemicarbazide (**3**), which proceeded with a yield of 83%, and the synthesis of the corresponding thiosemicarbazones **5a**–**k** of each hydroxylated benzaldehyde (**4a**–**e** and **4g**–**k**) or hydroxylated acetophenone (**4f**) proceeded with yields ranging from 60 to 70%. The synthesis of the final compounds **7a**–**k** by the Hantzsch heterocyclization of thiosemicarbazones **5a**–**k** with 4-chloroacetyl-catechol (**6**) proceeded with yields ranging from 40 to 59%, and the products **7a**–**k** were isolated as hydrochloride salts for improved long-term stability. The melting points of the final compounds were high due to the hydrochloride salt form and ranged from 243 °C to 288 °C. The identity and purity of the final compounds were confirmed using spectral analyses such as: high performance liquid chromatography (HPLC) coupled with mass spectrometry (MS), nuclear magnetic resonance (NMR), and infrared spectroscopy (IR). All graphical depictions of spectra recorded for each compound are illustrated in the Supplementary Materials.

### 2.2. Antiradical and Electron Transfer Assays

#### 2.2.1. Antiradical Assays

The antioxidant capacity of compounds **7a**–**k** was evaluated using DPPH^•^ and ABTS^•+^ radical scavenging assays. The resulting half-maximal inhibitory concentrations (IC_50_) are reported in Table 2, along with the corresponding values for the reference antioxidants, ascorbic acid, and Trolox.

#### 2.2.2. Electron Transfer Assays

The evaluation of the electron transfer capacity of compounds **7a**–**k** was conducted using four distinct methods: TAC, RP, FRAP, and CUPRAC, each involving different oxidizing agents and environmental conditions. The obtained results are presented in Table 3 and are expressed in molar equivalents (Eq) relative to the control (ascorbic acid and Trolox), calculated in accordance with Equation (2) described later in Materials and Methods.

### 2.3. In Silico Studies

#### 2.3.1. Theoretical Quantum Calculations

The frontier molecular orbitals play a crucial role in predicting the chemical behavior of a compound and were therefore calculated. The highest occupied molecular orbital (HOMO) reflects the molecule’s electron-donating capacity and its vulnerability to attack by electrophilic species, whereas the energy of the lowest unoccupied molecular orbital (LUMO) is associated with the molecule’s electron affinity and its susceptibility to nucleophilic attack. Literature reports suggest that the HOMO–LUMO energy gap may serve as an indicator of the antioxidant activity of the compounds, hence we calculated the energies of HOMO, LUMO, and the HOMO–LUMO gap, and the values obtained are presented in Table 4.

**Figure 1:**
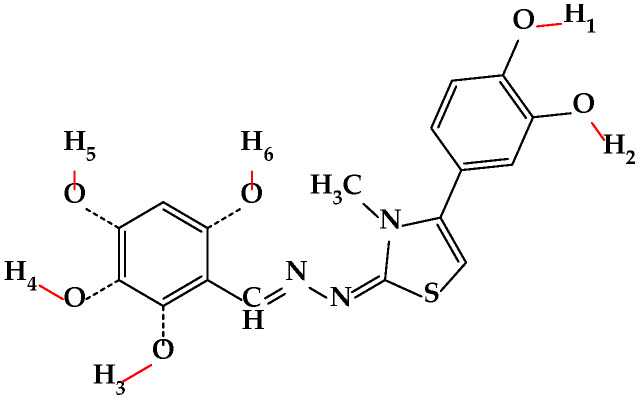
The general structure of the compounds, along with the potential sites (H_1_–H_6_) capable of releasing hydrogen atoms to neutralize radicals.

Furthermore, the bond dissociation enthalpy (BDE) for all phenol groups was calculated across all three environments. The molecular sites potentially releasing hydrogen atoms, identified as H_1_ to H_6_, are illustrated in the general structure shown in Figure 1. The calculated O-H bond dissociation enthalpies (BDE) for the H_1_–H_6_ sites in the studied compounds are provided in Table 5 (vacuum), Table 6 (nonpolar environment), and Table 7 (water).

#### 2.3.2. Molecular Properties with Influence on the Pharmacokinetics of Compounds

The physicochemical properties of the synthesized compounds were estimated using SwissADME. The most important parameters were evaluated to see if the compounds comply with Lipinski’s rule of 5 and to characterize their pharmacokinetic behavior in terms of oral bioavailability and permeability [25].

## 3. Discussion

### 3.1. Chemical Synthesis of the Compounds

The synthesis route followed was presented in Scheme 1 and was comprised of three sequential steps.

The first step in the synthesis route was the obtention of 4-methyl-3-thiosemicarbazide intermediate (compound **3**), from methyl isothiocyanate and hydrazine in ethanol. The characterization of the obtained intermediate was concordant to the data available (CAS 6610-29-3). The intermediate 4-methyl-3-thiosemicarbazide **3** was the common intermediate compound used for all future intermediates and final compounds.

The second step comprised the condensation between the intermediate 4-methyl-3-thiosemicarbazide **3** and various phenol derived carbonyl compounds **4a**–**k** in CH_3_COOH 10%, with the obtention of the intermediate 4-methyl thiosemicarbazones **5a**–**k**. The solvent used was chosen to ensure the solubilization of the starting compounds and proper precipitation of the resulting thiosemicarbazones **5a**–**k**, with low water solubility. The acetic acid was used to catalyze the reaction, by increasing the reaction rate thanks to the protonation of the carbonyl oxygen atom, to make it more electrophilic to be attacked easier by the nitrogen atom of 4-methyl-3-thiosemicarbazide (compound **2**).

In the third step, all 11 final compounds with a thiazolyl-polyphenolic structure were synthesized by applying the Hantzsch heterocyclization between intermediate thiosemicarbazones **5a**–**k** and 4-(chloroacetyl)catechol in acetone under reflux and were isolated as hydrochloride salts. All compounds were yellow or pale-yellow solid powders and were stored in tightly sealed brown bottles to prevent oxidation. The first confirmation of their identity was made by mass spectrometry, where the molecular peaks for each compound were identified and can be viewed in the Supplementary Materials Section, Figures S34–S44.

The structures of all analyzed compounds were confirmed by ^1^H-NMR spectroscopy (Supplementary Figures S12–S22). The thiazolyl-catechol scaffold was observed in all spectra, with aromatic signals appearing between 6 and 7 ppm. The thiazole H-5 proton typically resonated around 6.8 ppm and appeared as broad signal, although in some cases it overlapped with other benzylic or catechol protons. The catechol moiety exhibited three characteristic signals: a doublet of doublets around 6.8–7 ppm (*J*_1_ = 2 Hz, *J*_2_ = 8 Hz), and two additional signals between 6 and 7 ppm. These latter two signals sometimes overlapped, or one appeared as a doublet also around 7 ppm. The thiazole position 2 substituent, a benzylidene fragment bearing one, two, or three phenolic hydroxyl groups and occasionally methoxy or ethoxy substituents, also showed signals within the aromatic region (6–7 ppm). A singlet around 8.5 ppm, attributed to a benzylic proton, was consistently observed. In the aliphatic region, a singlet peak around 3.5 ppm, corresponding to the three protons from the N-methyl group of the thiazole ring, was present in all spectra. When methoxy or ethoxy groups were present on the benzylidene substituent, additional aliphatic signals were observed around 1.3 ppm for the ethoxy group and 3.8 ppm for methoxy and ethoxy groups.

Analysis of the ^13^C-NMR spectra (Supplementary Figures S23–S33) confirmed the presence of the expected carbon signals. The peaks of aromatic carbons were found within the characteristic range of 95–170 ppm. A single aliphatic carbon signal, present in all compounds, was observed around 35 ppm. Compounds containing methoxy or ethoxy substituents, as well as those derived from acetophenone, exhibited additional aliphatic signals between 30 and 60 ppm.

The IR spectral data obtained for compounds **7a**–**k** were consistent with their proposed structures, the synthesized compounds’ IR spectra (Supplementary Figures S1–S11) exhibiting the expected signals. Specifically, all compounds showed signals for phenolic OH groups in the 3000–3600 cm^−1^ region, C-N stretching around 1200 cm^−1^, and C=N stretching around 1600 cm^−1^. When present, methoxy and ethoxy groups were indicated by signals in the 1067–1333 cm^−1^ range, characteristic of aryl-alkyl ethers.

**Figure 2:**
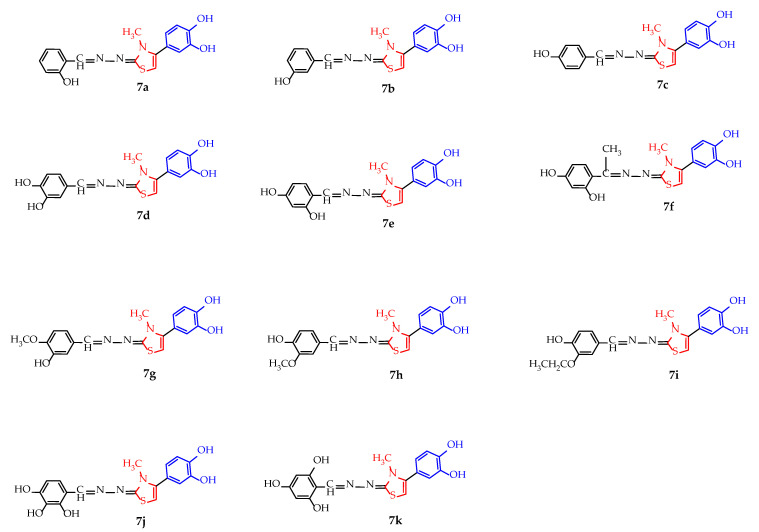
The chemical structure of the new N-methyl substituted thiazole derived polyphenols **7a**–**k**.

The structure of all **7a**–**k** compounds is presented in Figure 2.

Polyphenols, together with many other classes of compounds are originate products of the secondary metabolism of plants which play various roles for them [26,27,28]. However, isolating a specific compound from vegetal sources can be difficult, because hundreds of compounds can be found simultaneously, which makes isolation and purification difficult. Moreover, the plants can synthesize the desired substance in varying amounts, depending on various environmental factors. Providing a constant enough quantity of specific antioxidant compound or mixture or antioxidants from vegetal sources can be difficult. These shortcomings can be overcome by chemical synthesis of phenolic compounds of interest in the desired amount and high purity. The presented synthesis protocol has the advantage of using mild reaction conditions, having few reaction steps, and having a good atom economy overall.

### 3.2. Antiradical and Electron Transfer Assays

In this research, we performed six distinct tests to assess the antioxidant activity of the compounds **7a**–**k**, aiming to observe variations in their behavior based on the environmental factors, mechanism of action, and oxidizing agent strength. Their antiradical activity was evaluated using two assays, DPPH and ABTS. The main difference between these assays is represented by the environment, water for DPPH and ethanol for ABTS, where it is important to mention that the interaction of the solvent with the tested compounds might impact their antioxidant behavior. To further investigate the antioxidant behavior of the synthesized compounds under different conditions, we performed four electron transfer assays. The TAC assay uses Mo^+6^ as oxidizing agent and was conducted at 95 °C for 1.5 h. While both RP and FRAP assays use Fe^3+^ as oxidizing agent, they differed significantly in their conditions: RP was performed at 50 °C and pH = 6.6 for 20 min, whereas FRAP was conducted at room temperature and pH = 3.6 for 30 min. CUPRAC has Cu^2+^ as oxidizing agent in neutral pH value [29,30,31,32].

Using multiple assays for antioxidant research offers a series of advantages such as a comprehensive assessment of the antioxidant capacity under different conditions and various mechanisms which can lead to a more reliable evaluation. These complementary techniques provide validation and cross-verification of the results obtained through different mechanisms of action. This approach is important for understanding the interplay between the antioxidants and oxidants in the biological systems by using complementary assays to simulate the antioxidant behavior in various contexts.

#### 3.2.1. Antiradical Assays

According to the results of the DPPH scavenging assay illustrated in Table 2, compound **7j** had the lowest IC_50_ and is likely the most active compound in the series, probably due to its 2,3,4-trihydroxybenzylidene fragment which might confer a better capacity for neutralizing free radicals. The second most active compound is **7d**, whose free radical scavenging capacity is most likely due to the 3,4-dihydroxybenzylidene fragment. Even though compound **7k** has three hydroxyl groups like **7j**, their positions on the benzylidene fragment are not as favorable for neutralizing radicals as they are in **7j**. In comparison to the IC_50_ of ascorbic acid, all compounds exhibit greater activity, except for **7a**, which has a similar, though slightly higher IC_50_ value, thus indicating a lower activity. When compared to Trolox, compounds **7d**, **7e**, **7g**, **7i**, **7j**, and **7k** demonstrate superior activity, as evidenced by their lower IC_50_. Among all tested compounds and in comparison, to both reference substances, **7j** displays the highest activity.

In the ABTS scavenging assay, compound **7k**, which in the DPPH scavenging assay shown an intermediate activity, exhibited the lowest IC_50_ in this case. This can be attributed to various factors, such as the differences in the type of radical or environment, ethanol in DPPH assay, while ABTS was performed in water. This significant reduction in IC_50_ compared to Trolox is likely a consequence of the different reaction conditions, which appear to enhance the ABTS scavenging ability of **7k**. The second lowest IC_50_ was observed for compound **7e**, suggesting that the two hydroxyl phenolic groups, positioned similarly to those in compound **7k,** which had the lowest IC_50_, contribute significantly to its activity. Furthermore, compound **7j** showed excellent antioxidant activity, yielding the third lowest IC_50_. Compounds **7a** and **7h** displayed the highest IC_50_, indicating weaker antioxidant activity than the other synthesized compounds, yet still comparable to or better than that of Trolox, used as reference. The results summarized in Table 2 demonstrate that all synthesized compounds have a superior antiradical activity compared to Trolox.

#### 3.2.2. Electron Transfer Assays

According to the results illustrated in Table 3, in TAC assay, the highest electron transfer capacity is attributed to compound **7j**. Also, compound **7k** exhibited a very good electron transfer capacity compared to the other compounds in the series and ascorbic acid used as reference. Compounds **7d**, **7e**, and **7f**, having two OH groups on the benzylidene fragment, which contribute to the antioxidant activity, show good electron transfer capacity compared to the reference substance. Regarding the rest of the tested compounds, the values are quite close to 1, indicating that in this test, their antioxidant capacity is weaker. This is likely because they have only one OH group, and the methoxy and ethoxy groups present in compounds **7g**, **7h**, and **7i** do not significantly improve their electron transfer capacity.

According to the results obtained from the RP assay, illustrated in Table 3, all the tested compounds manifested higher antioxidant activity than ascorbic acid and Trolox, which were used as reference antioxidants. Compound **7j** has the 2,3,4-trihydroxybenzylidene fragment, which confers a strong capacity for transferring electrons to free radical species, followed by its isomer **7k**, which has the 2,4,6-trihydroxybenzylidene fragment, also contributing to the antioxidant activity. The difference between the two compounds is likely a consequence of the different positions of their OH groups and their impact on the electron transfer. Among the compounds **7a**, **7b**, and **7c**, the *meta* placement of the phenolic OH group seems to be optimal for electron transfer. This is further supported by the observation that within the series **7g**–**i**, which also contains a methoxy or ethoxy substituent, compound **7g**, possessing the *meta*-OH group, exhibits the highest activity, emphasizing the importance of this substitution pattern.

The FRAP assay further demonstrates the superior electron transfer capacity and excellent antioxidant behavior of both compounds **7j** and **7k**. Within the dihydroxy benzylidene series, compounds **7d**–**f** and **7e** exhibited the highest activity, likely due to the specific arrangement of its two phenolic OH groups. The difference in activity between **7e** and **7f** is attributed to the methyl substituent in **7f**, which may create steric hindrance, reducing the electron transfer susceptibility of the *ortho* OH group. As shown in Table 3, all synthesized compounds display an enhanced antioxidant activity related to Trolox.

The CUPRAC assay identified compound **7k** as exhibiting the highest activity, displaying the strongest electron transfer capacity compared to the Trolox reference. In this assay compound **7j** ranks second in activity, likely due to the different reaction medium and neutral pH. Compound **7e** is the third most active, and its strong antioxidant activity in this assay is probably a result of the phenolic OH groups in positions 2 and 4. For compound **7f**, which also possesses two OH groups in these positions, the methyl group in position 3 of the hydrazylidene fragment appears to sterically hinder the OH group in position 2, limiting its contribution to electron transfer. Among the series of compounds **7g**–**i**, each having one OH group and either a methoxy or ethoxy group, the presence of the ethoxy group in **7i** confers a significant advantage in terms of antioxidant activity. Finally, within the series of monohydroxy benzylidene compounds, **7a** exhibited the lowest electron transfer capacity, likely due to the specific position of its phenolic OH group.

### 3.3. In Silico Studies

#### 3.3.1. Theoretical Quantum Calculations

To establish structure–activity relationships and explain the results obtained in the performed antioxidant assays, we employed computational methods to investigate the influence of molecular structure on the antioxidant potential of these compounds. The highest occupied molecular orbital (HOMO) represents the molecule’s ability to donate electrons. A higher HOMO energy level signifies a greater predisposition for electron donation, thereby enhancing the compound’s capacity to neutralize free radicals. Conversely, the lowest unoccupied molecular orbital (LUMO) indicates the molecule’s ability to accept electrons. A lower LUMO energy level suggests a higher electron affinity, which can also contribute to the antioxidant activity. The HOMO–LUMO gap, the energy difference between these orbitals, is a key determinant of molecular reactivity. A smaller HOMO–LUMO gap implies a lower excitation energy, facilitating electron transfer and thus promoting antioxidant activity. Conversely, a larger gap indicates higher stability and reduced reactivity due to the greater energy required for electron transfer [33,34,35,36].

To characterize the antioxidant potential of compounds **7a**–**k**, we computed these electronic parameters in three distinct environments: vacuum, nonpolar medium, and water. This approach is aimed at comprehensively evaluating their antioxidant behavior under varying conditions. The data presented in Table 4 reveal a trend of decreasing HOMO–LUMO gap from vacuum to water. This observation suggests that the molecular stabilization varies across different media. Notably, water, being the closest physiological environment to the human body, may provide conditions that favor the antioxidant activity. The smaller HOMO–LUMO gaps observed in aqueous solution could therefore indicate a more effective action against free radicals associated with oxidative stress.

Compound **7f** exhibits the largest HOMO–LUMO gap values across all three evaluated environments indicating the lowest reactivity and, consequently, the weakest antioxidant potential within the series. Compared to compound **7e**, which demonstrates smaller gap values and thus higher reactivity, the observed difference between the two structures is most likely attributed to the presence of a methyl group on the hydrazone fragment from position 2 of the thiazole ring. This steric or electronic substitution influences the electron distribution and molecular stability, leading to an increase in the HOMO–LUMO gap and a diminished electron-donating capacity.

On the other hand, compound **7a** has the smallest HOMO–LUMO gap values in two of the studied media (water and nonpolar solvent) and exhibits a relatively small gap even in vacuum. This observation suggests a superior antioxidant potential for compound **7a**, as it is predisposed to a more facile electron transfer and, therefore, an enhanced capacity for neutralizing free radicals. This property is most likely a consequence of the presence of a phenolic OH group in the *ortho* position. The hydroxyl group, through its electron-donating effect, increases the electron density within the molecule and stabilizes the resulting radical cation after electron donation, thereby facilitating the free radical neutralization process.

Regarding BDE in the context of antioxidant activity, a lower BDE value indicates that O-H bonds are more readily cleaved, facilitating the donation of protons to neutralize the free radical species. Based on the data presented in Table 5, Table 6 and Table 7, as well as the electrostatic potential maps of each compound in Table S2 from the Supplementary Materials, the BDEs of the phenol OH groups on the catechol moiety in position 4 of the thiazole ring are not significantly influenced by the substituents from position 2. Consequently, O-H_1_ consistently exhibits similar and lower BDE values compared to O-H_2_ across all compounds and environments, suggesting that O-H_1_ more readily donates its proton, irrespective of the surrounding environment. Comparing BDEs across the three environments, O-H bond cleavage is thermodynamically most favorable (requires the least energy) in vacuum, slightly more difficult in nonpolar environment and least favorable in water, finding which is in accordance with literature reports analyzing polyphenolic antioxidants. The conclusion is based on the lack of charge of the radical in the radical transfer of hydrogen, favored by a less polar environment. The solvent effect of water on the HAT mechanism when compared to gas phase increases the BDE with approximately 2 kcal/mol for each OH bond. Therefore SPLET mechanism could be applied to be more precise in context of a polar solvent, to analyze the energy gap in the intermediate steps, but it would be too far taking into account the scope of the present research, taking into account the possible intermediates and pH dependance of the SPLET mechanism [33,37,38,39].

The choice of analyzing only the HAT mechanism for the current molecules was thanks to its wide use in various fields, with high accuracy and computational cheap [40,41] and disregarding the mechanism involved, the BDE describes the antiradical activity of polyphenols [41,42]. Overall, the BDE values are relatively low, indicating that these compounds can readily donate protons and effectively exhibit antioxidant activity.

Analyzing the BDE values for O-H_3_, O-H_4_, O-H_5_, and O-H_6_ (where present), these values are generally higher, suggesting a less facile proton donation compared to the OH groups from the catechol fragment. Focusing on the substituent in position 2 of the thiazole ring, it was observed that for the monohydroxy compounds (**7a**–**c**), the BDE is influenced by the OH group’s position. Compound **7c** exhibits the lowest BDE values in all cases, a phenomenon potentially explained by electron delocalization through resonance and stabilization of the resulting radical after proton donation, thus favoring a decrease in BDE. The values obtained for O-H_3_ are the highest in all cases, most likely due to its *ortho* position, which predisposes it to intramolecular hydrogen bond formation. Comparing the BDE values of O-H_5_, which is present in many molecules, we observed that compound **7d** exhibits the lowest value, likely attributable to the presence of a second OH group in the adjacent position, forming a catechol fragment, which is known to act as a potent antioxidant. It can be observed that the presence of electron-donating methoxy and ethoxy groups in compounds **7i** and **7h** leads to a decrease in the BDE value for O-H_5_, thus favoring proton donation.

The compounds were ranked based on their antioxidant strength by considering the lowest BDE for each, as this value reflects the ease of hydrogen donation. In vacuum, the compound with the lowest BDE, **7d** (64.58 kcal/mol), is the strongest antioxidant since it requires the least energy to break the O-H bond. On the other hand, compounds with higher BDE values, such as **7a** (BDE = 69.23 kcal/mol), are weaker antioxidants because their O-H bonds are harder to break, making them less efficient at hydrogen donation. Therefore, the antioxidant activity in vacuum decreases in the following order: **7d**, **7c**, **7g**, **7i**, **7e**, **7h**, **7b**, **7j**, **7k**, **7f**, and **7a**.

Among the compounds, in the nonpolar environment, **7d** exhibited the lowest BDE (65.31 kcal/mol), indicating it has the strongest antioxidant potential. In contrast, **7a** had the highest BDE (69.38 kcal/mol), making it the weakest antioxidant in this set, as its O-H bond is more resistant to dissociation. The compounds were ranked in descending order of antioxidant activity in nonpolar environment as follows: **7d**, **7g**, **7c**, **7f**, **7j**, **7e**, **7i**, **7h**, **7k**, **7b**, and **7a**.

In water, **7d** exhibited the lowest BDE (67.68 kcal/mol), making it the strongest antioxidant, as it requires the least energy to break the O-H bond. In contrast, **7b** had the highest BDE (71.79 kcal/mol), making it the weakest antioxidant in this set, as its O-H bond is more resistant to dissociation. Therefore, the compounds were ranked in descending order of antioxidant strength as follows: **7d**, **7j**, **7f**, **7c**, **7g**, **7i**, **7e**, **7h**, **7k**, **7a**, and **7b**, with lower BDE values representing a stronger antioxidant potential due to easier hydrogen donation in water.

#### 3.3.2. Molecular Properties with Influence on the Pharmacokinetics of Compounds

The physicochemical properties of the compounds **7a**–**k** obtained in silico using SwissADME are presented in Table 8. According to these results, compounds **7a**–**k** complied with Lipinski’s rule of 5, with zero violations. The molecular weights of all compounds are below 500, meaning they adhere to Lipinski’s rule regarding the maximum allowed molecular weight, ranging from 341 to 385. Furthermore, another Lipinski rule states that the number of rotatable bonds should be below 10 and the compounds have a number between 3 and 5, which means they also comply with this rule. The number of hydrogen bond donors and acceptors varies from one compound to another. The number of hydrogen bond donors ranges from 3 to 5, depending on the number of phenolic OH groups present in the molecule, while the number of hydrogen bond acceptors varies depending on the nature of the substituent in position 2 of the thiazole. The TPSA values are constant for compounds with the same number of OH groups and increase with the increasing number of OH groups or with the presence of methoxy and ethoxy groups in the molecule. The lipophilicity-hydrophilicity balance is given by MlogP, which varies from one compound to another depending on the number of OH groups, which are a determining factor regarding a compound’s lipophobicity. The more hydroxyl groups there are, the lower the MlogP will be, indicating that the compound is more hydrophilic. According to the data illustrated in Table 8, all compounds had moderate to good water solubility.

## 4. Materials and Methods

### 4.1. Materials

#### 4.1.1. Chemicals and Consumables

All chemicals employed in the synthesis, purification, structural analysis, and antioxidant activity evaluation were of the appropriate purity grade, tailored to the requirements of each process. These chemicals were obtained from authorized local suppliers. The following chemicals: methyl isothiocyanate, hydrazine monohydrate 98%, benzaldehydes, and 4-chloro-acetyl-catechol used in the synthesis of compounds **7a**–**k**, were sourced from Sigma-Aldrich, Merck (Merck KGaA, Darmstadt, Germany), Alfa Aesar (Thermo Scientific, Waltham, MA, USA), and TCI (Tokyo Chemical Industry UK Ltd., Oxford, UK). Dimethyl sulfoxide (DMSO) (≥99%) was obtained from Merck (Merck KGaA, Darmstadt, Germany) for the preparation of solutions of compounds **7a**–**k**.

#### 4.1.2. Instruments

The synthesized compounds were characterized using a suite of physicochemical and spectroscopic techniques. Melting points were determined using an MPM-H1 melting point apparatus (Schorpp Gerätetechnik, Überlingen, Germany) employing the glass capillary method. Mass spectra were acquired in negative ionization mode using an Agilent 1100 series system coupled with an Agilent Ion Trap SL mass spectrometer (Agilent Technologies, Santa Clara, CA, USA). Infrared spectra were recorded on a Jasco FT/IR 6100 spectrometer (Jasco, Cremella, Italy) using KBr pellets prepared under vacuum. ^1^H and ^13^C NMR spectra were obtained on a Bruker Avance spectrometer (Bruker, Karlsruhe, Germany) with samples dissolved in dimethyl sulfoxide-*d*_6_ (DMSO-*d*_6_). Chemical shifts were referenced to the residual solvent peak, with tetramethylsilane (TMS) used for calibration. All NMR spectra exhibited signals in the expected chemical shift regions, displaying the anticipated intensities and multiplicities, which were assigned to the corresponding structural moieties (*s*—singlet; *d*—doublet; *dd*—doublet of doublets; *t*—triplet; *m*—multiplet). Specific abbreviations were employed to indicate the location of hydrogen or carbon atoms within the molecule: ctc—catechol fragment from position 4 of the thiazole, Bz—for benzylidene fragment, Th—for thiazole.

To assess the antiradical and electron transfer assays, the absorbance of the resulting mixtures was measured spectrophotometrically using a Specord 210 PLUS UV-VIS spectrometer (Analytik Jena AG, Jena, Germany) in low-volume, single-use 10 mm plastic cuvettes. Density functional theory (DFT) calculations were conducted on a system equipped with an Intel Core i7-12700KF processor running Windows 10 (Microsoft, Redmond, WA, USA).

### 4.2. Methodology for Synthesis and Characterization of Final Compounds

For the synthesis of compounds **7a**–**k**, we used methyl isothiocyanate (**1**) as a starting material and synthesized a stock quantity of 4-methyl-3-thiosemicarbazide (**3**). Thus, in a glass flask, 90 mmol of methyl isothiocyanate (**1**) were dissolved in 15 mL of 96% ethanol, to which 90 mmol of 98% hydrazine monohydrate (**2**) were added dropwise. The reaction occurred almost instantly under cold conditions with continuous stirring and was maintained for 0.5 h to ensure a quantitative reaction. The resulting precipitate of 4-methyl-3-thiosemicarbazide (**3**) was filtered under vacuum, purified using 96% ethanol, and properly stored for use in subsequent steps. The reaction was monitored by thin layer chromatography (TLC), and the reaction yield was 83%. The next step involved the synthesis of thiosemicarbazones (**5**) by condensing 4-methyl-3-thiosemicarbazide (**3**) with various hydroxylated benzaldehydes and acetophenones (**4**) in water containing 10% glacial acetic acid [43]. We started with 4 mmol of 4-methyl-3-thiosemicarbazide (**3**), dissolved in 5 mL of an aqueous solution of 10% glacial acetic acid, to which 4 mmol of hydroxylated benzaldehyde/acetophenone (**4**) were added. The mixture was kept under reflux with continuous stirring for 3 h, and filtration was carried out while hot, to isolate the resulting precipitate [24]. Due to the instability of the obtained thiosemicarbazones (**5**) over time, they were kept in a dry atmosphere with anhydrous MgSO_4_ in vacuum for 3 h to remove residual moisture. From the dried material, 2 mmol were taken and dissolved in 3 mL of acetone, to which 2 mmol of 4-chloro-acetyl-catechol (**6**) was added, and was refluxed for 2–3 h [44]. Filtration was performed while hot to avoid contamination of the final product with cold-insoluble impurities. All steps were monitored by TLC and are illustrated in Scheme 1.

All final compounds were isolated as hydrochloride salts to improve their long-term stability and ease of solubilization in the solvents used for the antioxidant assays. The compounds were analyzed using well-established spectral techniques, including MS, IR, and NMR.

*4-((E)-2-(((E)-2-hydroxybenzylidene)hydrazineylidene)-3-methyl-2,3-dihydrothiazol-4-yl)benzene-1,2-diol* (**7a**): pale yellow solid; mp = 243 °C; yield = 58%; FT IT (KBr) v_max_ cm^−1^: 1272 (N-CH_3_), 1602 (C=N), 2951,3118, 3334 (phenolic OH); MS: *m/z* = 340.8; ^1^H-NMR (DMSO-*d*_6_, 500 MHz) δ: 3.47 (*s*, 3H, -CH_3_), 6.72 (*s*, 1H, Th), 6.79 (*dd*, 1H, ctc, *J*_1_ = 2 Hz, *J*_2_ = 8.5 Hz), 6.89 (*t*, 2H, ctc, *J* = 2 Hz), 6.90–6.93 (*m*, 1H, Bz), 6.97 (*dd*, 1H, Bz, *J*_1_ = 1 Hz, *J*_2_ = 8.5 Hz), 7.29–7.32 (*m*, 1H, Bz), 7.62 (*dd*, 1H, Bz, *J*_1_ = 1,5 Hz, *J*_2_ = 7.5 Hz), 8.82 (*s*, 1H, Bz); ^13^C-NMR (DMSO-*d*_6_, 125 MHz) δ: 35.0 (-CH_3_), 102.0 (TH-C_5_), 115.9 (ctc), 116.4 (ctc), 116.6 (Bz), 119.2 (Bz), 119.6 (ctc), 120.7 (Bz), 127.9 (ctc), 130.6 (Bz), 131.8 (Bz), 142.2 (Th-C_4_), 145.6 (ctc-OH), 147.2 (ctc-OH), 149.9 (Bz), 157.5 (Bz-OH), 168.1 (Th-C_2_).

*4-((E)-2-(((E)-3-hydroxybenzylidene)hydrazineylidene)-3-methyl-2,3-dihydrothiazol-4-yl)benzene-1,2-diol* (**7b**): pale yellow solid; mp = 253 °C; yield = 51%; FT IT (KBr) v_max_ cm^−1^: 1273 (n-CH_3_), 1606 (C=N), 3095, 3237, 3345 (phenolic OH); MS: *m/z* = 340.4; ^1^H-NMR (DMSO-*d*_6_, 500 MHz) δ: 3.57 (*s*, 3H, -CH_3_), 6.79 (*dd*, 1H, ctc, *J*_1_ = 2 Hz, *J*_2_ = 8 Hz), 6.90–6.92 (*m*, 4H, Bz + Th + ctc), 7.14 (*d*, 1H, Bz, *J* = 8 Hz), 7.20 (*s*, 1H, Bz), 7.28 (*t*, 1H, Bz, *J* = 8 Hz), 8.67 (*s*, 1H, Bz); ^13^C-NMR (DMSO-*d*_6_, 125 MHz) δ: 35.7 (-CH_3_), 104.5 (Th-C_5_), 112.9 (Bz), 115.9 (ctc), 116.7 (ctc), 118.3 (Bz), 118.9 (ctc), 119.2 (Bz), 120.8 (Bz), 130.0 (ctc), 134.3 (Th-C_4_), 142.7 (Bz), 145.5 (ctc-OH), 147.2 (ctc-OH), 150.8 (Bz), 157.8 (Bz-OH), 168.3 (Th-C_2_).

*4-((E)-2-(((E)-4-hydroxybenzylidene)hydrazineylidene)-3-methyl-2,3-dihydrothiazol-4-yl)benzene-1,2-diol* (**7c**): pale yellow solid; mp = 262 °C; yield = 58%; FT IR (KBr) v_max_ cm^−1^: 1272 (N-CH_3_), 1602 (C=N), 3123, 3215, 3411 (phenolic *OH*); MS: *m/z* = 340.9; ^1^H-NMR (DMSO-*d*_6_, 500 MHz) δ: 3.52 (*s*, 3H, -CH_3_), 6.79 (*dd*, 1H, ctc, *J*_1_ = 2.5 Hz, *J*_2_ = 8 Hz), 6.85 (*s*, 1H, Th), 6.88–6.90 (*m*, 4H, Bz + ctc), 7.60 (*d*, 2H, Bz, *J* = 9 Hz), 8.55 (*s*, 1H, Bz); ^13^C-NMR (DMSO-*d*_6_, 125 MHz) δ: 35.0 (-CH_3_), 104.1 (Th-C_5_), 115.5 (Bz), 116.2 (ctc), 119.3 (Bz), 120.4 (ctc), 123.8 (ctc), 128.9 (Bz), 142.1 (Th-C_4_), 145.1 (ctc-OH), 146.8 (ctc-OH), 150.5 (Bz), 159.9 (Bz-OH), 167.3 (Th-C_2_).

*4-((E)-2-(((E)-3,4-dihydroxybenzylidene)hydrazineylidene)-3-methyl-2,3-dihydrothiazol-4-yl)benzene-1,2-diol* (**7d**): pale yellow solid; mp = 269 °C; yield = 59%; FT IT (KBr) v_max_ cm^−1^: 1275 (N-CH_3_), 1602 (C=N), 3019, 3122, 3243, 3398 (phenolic OH); MS: *m/z* = 356.6; ^1^H-NMR (DMSO-*d*_6_, 500 MHz) δ: 3.57 (*s*, 3H, -CH_3_), 6.79 (*dd*, 1H, ctc, *J*_1_ = 2 Hz, *J*_2_ = 8.5 Hz), 6.86 (*d*, 1H, Bz, *J* = 8 Hz), 6.89–6.92 (*m*, 3H, Bz + Th + ctc), 7.01 (*dd*, 1H, ctc, *J*_1_ = 2 Hz, *J*_2_ = 8 Hz), 7.24 (*d*, 1H, Bz, *J* = 2 Hz), 8.57 (*s*, 1H, Bz); ^13^C-NMR (DMSO-d6, 125 MHz) δ: 35.7 (-CH_3_), 104.4 (Th-C_5_), 112.9 (ctc), 115.8 (ctc), 115.9 (Bz), 116.7 (Bz), 119.2 (Bz), 120.8 (ctc), 121.3 (Bz), 124.3 (ctc), 142.7 (Th-C_4_), 145.5 (Bz-OH), 145.8 (ctc-OH), 147.3 (ctc-OH), 149.1 (Bz-OH), 151.3 (Bz), 167.5 (Th-C_2_).

*4-((E)-2-(((E)-2,4-dihydroxybenzylidene)hydrazineylidene)-3-methyl-2,3-dihydrothiazol-4-yl)benzene-1,2-diol* (**7e**): yellow solid; mp = 261 °C; yield = 52%; FT IT (KBr) v_max_ cm^−1^: 1213 (N-CH_3_), 1611 (C=N), 2998, 3127, 3317, 3526 (phenolic OH); MS: *m/z* = 356.3; ^1^H-NMR (DMSO-*d*_6_, 500 MHz) δ: 3.43 (*s*, 3H, -CH_3_), 6.36–6.39 (*m*, 2H, Bz), 6.67 (*s*, 1H, Th), 6.78 (*dd*, 1H, ctc, *J*_1_ = 2.5 Hz, *J*_2_ = 8 Hz), 6.86–6.88 (*m*, 2H, Bz + ctc), 7.43 (*d*, 1H, ctc, *J* = 8 Hz); ^13^C-NMR (DMSO-d6, 125 MHz) δ: 34.6 (-CH_3_), 102.4 (Bz), 108.0 (Th-C_5_), 110.7 (Bz), 112.3 (Bz), 115.7 (ctc), 116.4 (ctc), 119.9 (ctc), 120.5 (ctc), 129.3 (Bz), 142.2 (Th-C_4_), 154.4 (ctc-OH), 147.0 (ctc-OH), 157.1 (Bz-OH), 159.2 (Bz-OH), 161.1 (Bz), 166.9 (Th-C_2_).

*4-((E)-2-(((E)-1-(2,4-dihydroxyphenyl)ethylidene)hydrazineylidene)-3-methyl-2,3-dihydrothiazol-4-yl)benzene-1,2-diol* (**7f**): yellow solid; mp = 267 °C; yield = 45%; FT IT (KBr) v_max_ cm^−1^: 1274 (N-CH_3_), 1590 (C=N), 3032, 3091, 3321, 3474 (phenolic OH); MS: *m/z* = 370.3; ^1^H-NMR (DMSO-*d*_6_, 500 MHz) δ: 2.47 (*s*, 3H, Bz-CH_3_), 3.32 (*s*, 3H, -CH_3_), 6.27 (*s*, 1H, Bz), 6.29 (*d*, 1H, Bz, *J* = 2.5 Hz), 6.35 (*dd*, 1H, ctc, *J*_1_ = 2.5 Hz, *J*_2_ = 4 Hz), 6.76 (*dd*, 1H, ctc, *J*_1_ = 2 Hz, *J*_2_ = 8 Hz), 6.85 (*t*, 2H, Bz + Th, *J* = 2.5 Hz), 7.40 (*d*, 1H, ctc, *J* = 9 Hz) ^13^C-NMR (DMSO-d6, 125 MHz) δ: 14.0 (-OCH_3_), 33.6 (-CH_3_), 96.9 (Bz), 102.9 (Th-C_5_), 107.1 (Bz), 112.1 (ctc), 115.9 (Bz), 116.3 (ctc), 120.3 (ctc), 121.2 (ctc), 129.6 (Bz), 141.7 (Th-C_4_), 145.5 (ctc-OH), 146.7 (ctc-OH), 159.9 (Bz-OH), 160.2 (Bz-OH), 160.6 (Bz), 165.9 (Th-C_2_).

*4-((E)-2-(((E)-3-hydroxy-4-methoxybenzylidene)hydrazineylidene)-3-methyl-2,3-dihydrothiazol-4-yl)benzene-1,2-diol* (**7g**): pale yellow solid; mp = 256 °C; yield = 55%; FT IT (KBr) v_max_ cm^−1^: 1067, 1094 (OCH_3_), 1279 (N-CH_3_), 1607 (C=N), 3126, 3412, 3557 (phenolic OH); MS: *m/z* = 370.3; ^1^H-NMR (DMSO-*d*_6_, 500 MHz) δ: 3.49 (*s*, 3H, -CH_3_), 3.83 (*s*, 3H, -OCH_3_), 6.79 (*dd*, 2H, ctc, *J*_1_ = 2 Hz, *J*_2_ = 8 Hz), 6.87 (*s*, 1H, Th), 7.01 (*d*, 1H, Bz, *J* = 8 Hz), 7.12 (*dd*, 1H, Bz, *J*_1_ = 2 Hz, *J*_2_ = 8 Hz), 7.27 (*d*, 1H, Bz, *J* = 2 Hz), 8.47 (*s*, 1H, Bz); ^13^C-NMR (DMSO-d6, 125 MHz) δ: 35.3 (-CH_3_), 55.8 (-OCH_3_), 108.1 (Th-C_5_), 112.0 (ctc), 112.4 (Bz), 115.9 (ctc), 116.7 (Bz), 120.8 (ctc), 121.1 (Bz), 125.8 (ctc), 135.7 (Bz), 143.1 (Th-C_4_), 147.0 (ctc-OH), 147.2 (ctc-OH), 150.9 (Bz), 153.1 (Bz), 168.0 (Th-C_2_).

*4-((E)-2-(((E)-4-hydroxy-3-methoxybenzylidene)hydrazineylidene)-3-methyl-2,3-dihydrothiazol-4-yl)benzene-1,2-diol* (**7h**): pale yellow solid; mp = 250 °C; yield = 50%; FT IT (KBr) v_max_ cm^−1^: 1049, 1118 (-OCH_3_), 1267 (N-CH_3_), 1601 (C=N), 3115, 3196, 3513 (phenolic OH); MS: *m/z* = 371.2; ^1^H-NMR (DMSO-*d*_6_, 500 MHz) δ: 3.54 (*s*, 3H, -CH_3_), 3.84 (*s*, 3H, -OCH_3_), 6.79 (*dd*, 1H, ctc, *J*_1_ = 2 Hz, *J*_2_ = 8.5 Hz), 6.84 (*s*, 1H, Th), 6.88–6.91 (*m*, 3H, Bz + ctc), 7.20 (*dd*, 1H, Bz, *J*_1_ = 2 Hz, *J*_2_ = 8.5 Hz), 7.31 (*d*, 1H, Bz, *J* = 2 Hz), 8.56 (*s*, 1H, Bz); ^13^C-NMR (DMSO-d6, 125 MHz) δ: 35.5 (-CH_3_), 55.5 (-OCH_3_), 103.9 (Th-C_5_), 109.9 (Bz), 115.7 (ctc), 115.8 (Bz), 116.6 (ctc), 119.4 (Bz), 120.7 (ctc), 122.0 (Bz), 124.6 (ctc), 142.5 (Th-C_4_), 145.4 (ctc-OH), 147.1 (ctc-OH), 148.0 (Bz), 149.7 (Bz-OH), 150.9 (Bz), 167.8 (Th-C_2_).

*4-((E)-2-(((E)-3-ethoxy-4-hydroxybenzylidene)hydrazineylidene)-3-methyl-2,3-dihydrothiazol-4-yl)benzene-1,2-diol* (**7i**): pale yellow solid; mp = 251 °C; yield = 50%; FT IT (KBr) v_max_ cm^−1^: 1105, 1117 (-OCH_2_CH_3_), 1275 (N-CH_3_), 1602 (C=N), 3045, 3163, 3385 (phenolic OH); MS: *m/z* = 385.0; ^1^H-NMR (DMSO-*d*_6_, 500 MHz) δ: 1.37 (*t*, 3H, -OCH_2_CH_3_, *J* = 7 Hz), 3,52 (*s*, 3H, -CH_3_), 4.07 (*d*, 1H, -OCH_2_CH_3_, *J* = 7 Hz), 4.10 (*d*, 1H, -OCH_2_CH_3_, *J* = 6.5 Hz), 6.79 (*dd*, 1H, ctc, *J*_1_ = 2 Hz, *J*_2_ = 8.5 Hz), 6.82 (*s*, 1H, Th), 6.88–6.91 (*m*, 3H, Bz + ctc), 7.19 (*dd*, 1H, Bz, *J*_1_ = 2 Hz, *J*_2_ = 8.5 Hz), 7.30 (*d*, 1H, Bz, *J* = 2 Hz), 8.51 (*s*, 1H, Bz); ^13^C-NMR (DMSO-d6, 125 MHz) δ: 14.8 (-OCH_2_CH_3_), 35.5 (-CH_3_), 64.0 (-OCH_2_CH_3_), 103.9 (Th-C_5_), 111.4 (Bz), 115.9 (ctc), 116.7 (ctc), 119.7 (Bz), 120.8 (ctc), 122.1 (Bz), 125.8 (ctc), 130.5 (Bz), 142.6 (Th-C_4_), 145.6 (ctc-OH), 146.6 (Bz), 147.3 (ctc-OH), 150.1 (Bz), 151.0 (Bz-OH), 167.9 (Th-C_2_).

*4-((E)-(((E)-4-(3,4-dihydroxyphenyl)-3-methylthiazol-2(3H)-ylidene)hydrazineylidene)methyl)benzene-1,2,3-triol* (**7j**): yellow solid; mp = 263 °C; yield = 40%; FT IT (KBr) v_max_ cm^−1^: 1255 (N-CH_3_), 1611 (C=N), 3075, 3143, 3166, 3245, 3506 (phenolic OH); MS: *m/z* = 372.2; ^1^H-NMR (DMSO-*d*_6_, 500 MHz) δ: 3,44 (*s*, 3H, -CH_3_), 6.43 (*d*, 1H, Bz, *J* = 8 Hz), 6.68 (*s*, 1H, Th), 6.77 (*dd*, 1H, ctc, *J*_1_ = 2 Hz, *J*_2_ = 8 Hz), 6.87–6.88 (*m*, 2H, Bz + ctc), 6.96 (*d*, 1H, ctc, *J* = 8.5 Hz) 8.68 (*s*, 1H, Bz); ^13^C-NMR (DMSO-d6, 125 MHz) δ: 34.8 (-CH_3_), 104.3 (Bz), 108.2 (Th-C_5_), 111.6 (Bz), 115.9 (ctc), 116.6 (ctc), 119.1 (Bz), 120.0 (ctc), 120.7 (ctc), 132.9 (Bz), 142.4 (Th-C_4_), 145.6 (ctc-OH), 147.2 (Bz-OH). 147.6 (ctc-OH), 149.2 (Bz-OH), 151.3 (Bz), 167.0 (Th-C_2_).

*2-((E)-(((E)-4-(3,4-dihydroxyphenyl)-3-methylthiazol-2(3H)-ylidene)hydrazineylidene)methyl)benzene-1,3,5-triol* (**7k**): yellow solid; mp = 288 °C; yield = 40%; FT IT (KBr) v_max_ cm^−1^: 1239 (N-CH_3_), 1607 (C=N), 3021, 3114, 3265, 3324, _3428_ (phenolic OH); MS: *m/z* = 372.1; ^1^H-NMR (DMSO-*d*_6_, 500 MHz) δ: 3,45 (*s*, 3H, -CH_3_), 5.93 (*s*, 2H, Bz), 6.65 (*s*, 1H, Th), 6.77 (*dd*, 1H, ctc, *J*_1_ = 2 Hz, *J*_2_ = 8 Hz), 6.87–6.89 (*m*, 2H, ctc), 8.89 (*s*, 1H, Bz); ^13^C-NMR (DMSO-d6, 125 MHz) δ: 34.9 (-CH_3_), 94.7 (Bz), 99.1 (Bz), 101.2 (Th-C_5_), 116.0 (ctc), 116.8 (ctc), 120.0 (ctc), 120.7 (ctc), 142.6 (Th-C_4_), 145.6 (ctc-OH), 147.2 (ctc-OH), 150.3 (Bz), 159.9 (Bz-OH), 162.4 (Bz-OH), 166.1 (Th-C_2_).

### 4.3. Antioxidant Assays

Stock solutions (2 mM) of the compounds **7a**–**k** and reference antioxidants (ascorbic acid and Trolox) were prepared by dissolving the solid compounds in DMSO. Further dilution with DMSO yielded a second set of solutions at 0.2 mM concentration. Absorption spectra of the tested compounds (400–800 nm) confirmed the absence of significant absorption at the wavelengths used in the assays. All measurements were performed against appropriate blank samples. The antioxidant activity of compounds **7a**–**k** was quantified by calculating the half-maximal inhibitory concentration (IC_50_) using Equation (1) for the radical scavenging assays (DPPH and ABTS), and the molar equivalent activity using Equation (2) for the electron transfer assays (TAC, RP, FRAP, and CUPRAC).

(1) $$radical\;scavenging(\%)=\frac{control\;absorbance-sample\;absorbance}{control\;absorbance}\times 100$$

(2) $$equivalents\;of\;control=\frac{sample\;absorbance}{control\;absorbance}$$

#### 4.3.1. Antiradical Assays

The DPPH^•^ radical-scavenging assay, originally described by Brand-Williams et al., measures the ability of an antioxidant to donate a hydrogen atom to the stable violet DPPH^•^ radical, converting it to a non-radical yellow compound. The decrease in DPPH^•^ absorbance at 517 nm is proportional to the amount of radical neutralized. For the assay, 25, 50, 100, 125, and 150 µL of each 0.2 mM sample solution were diluted with DMSO to a consistent final volume, then mixed with 1 mL of DPPH^•^ reagent and incubated in the dark for 30 min, as previously reported [15,45,46,47].

The ABTS^•+^ decolorization assay, based on the method of Re et al. [48] and conducted as previously described by our group [15,29], assessed the antioxidant activity by monitoring the reduction of the green ABTS^•+^ radical to its colorless form. The stability of the ABTS^•+^ reagent (λ = 734 nm) in 0.1 M potassium phosphate buffer (pH = 7.4) was confirmed over 1 h. The ABTS^•+^ radical was generated by overnight activation with MnO_2_. For each assay, 100 μL of the 0.2 mM sample solutions (up to 200 μL) were mixed with 2000 μL of ABTS^•+^ reagent, shaken vigorously for 10 min at room temperature in the dark, and the absorbance was measured at 734 nm [29,49].

#### 4.3.2. Electron Transfer Assays

The total antioxidant capacity (TAC) assay was performed following the methodology described in our previous publication, which was grounded in initial literature reports [15,29,50]. In this assay 10 mL of the reagent (containing 0.6 M H_2_SO_4_, 28 mM Na_3_PO_4_, and 4 mM (NH_4_)_6_Mo_7_O_24_) was mixed with 1000 μL of each compound and standard solution (derived from 2 mM stock solutions). The mixtures were incubated in sealed glass tubes at 95 °C for 90 min and cooled to room temperature. From each of them, 1000 µL were diluted with 1000 μL of water, and the absorbance was measured at 695 nm.

The reducing power (RP) assay, adapted from the established methods [15,29,49], assesses the ability of the tested compounds to reduce ferric ions (Fe^3+^) in potassium ferricyanide to ferrocyanide, forming a blue complex. Briefly, 1000 μL of each compound and standard solution (from 0.2 mM stock solutions) was mixed with 400 μL of phosphate buffer (0.2 M, pH = 6.6) and 400 μL of 1% (*w*/*v*) K_3_[Fe(CN)_6_]. After incubation in sealed tubes at 50 °C for 20 min and cooling to room temperature, 500 μL of 10% (*w*/*w*) trichloroacetic acid was added. Following a 30 min incubation at room temperature, 250 μL of each mixture was combined with 140 μL of 0.1% (*w*/*v*) FeCl_3_ and 1000 μL of distilled water, and the absorbance was measured at 695 nm.

The ferric-reducing antioxidant potential (FRAP) assay is based on the transfer of an electron from the antioxidant under examination to Fe^3+^, converting it into Fe^2+^. The resulting Fe^2+^ ions are then chelated by 2,4,6-tripyridyl-s-triazine, forming a blue-colored complex that exhibits an absorption maximum at λ = 593 nm [29,51]. In this assay, 500 µL of 0.2 mM solutions of the compounds and reference compounds were combined with 600 µL of FRAP reagent and 1200 µL of acetate buffer (0.3 M, pH = 3.6), in accordance with prior reports [15,29]. The solutions were mixed thoroughly in the dark, and their absorbance was measured spectrophotometrically.

Using the CUPRAC (CUPric-reducing antioxidant capacity) method, the electron-donating capacity of the compounds was evaluated. This assay protocol is a modification of the methods described by Alam et al., Özyürek et al., and Apak et al. [29,52,53]. In this assay, 250 µL of 10 mM CuCl_2_, 1 mL of 1 M ammonium acetate buffer, and 250 µL of 7.5 mM neocuproine in ethanol were mixed with 125 µL of the sample and reference compound solutions (0.2 mM). The mixtures were shaken for 30 min in the dark, after which their absorbance was determined spectrophotometrically at λ = 450 nm.

### 4.4. In Silico Studies

#### 4.4.1. Theoretical Quantum Calculation

Two primary mechanisms were considered to characterize the potential antioxidant activities of compounds **7a**–**k**: one involving electron transfer, analyzed via frontier molecular orbitals—specifically, the energy levels of the highest occupied molecular orbital (HOMO) and the lowest unoccupied molecular orbital (LUMO), and the other involving hydrogen atom transfer (HAT) via heterolytic cleavage of the OH bond (Ar-OH → Ar-O^•^ + H^•^). Literature suggests that HAT mechanism is preferred for phenolic molecules, for example, where an external radical is involved, such as DPPH [54]. The O-H bond dissociation energy (BDE) was calculated using the formula BDE = H(ArO^•^) + H(H^•^) − H(ArOH) [33,34,36,55,56].

The computational analyses for these compounds were conducted using Spartan24 (Wavefunction, CA, USA) at the B3LYP level of theory with a 6-311+G(d,p) basis set in vacuum, nonpolar solvent (ε = 7.43), and water (ε = 78.3) environments. This approach aimed to examine how different solvents might impact the antioxidant properties of the studied molecules, employing the polarizable continuum solvation model. All calculations were performed at 298.15K and 1 atm. The geometry optimizations of the molecules were executed using the built-in Pulay Direct Inversion in the Iterative Subspace (DIIS) method, in conjunction with geometric direct minimization [33,35,36]. For compounds **7a**–**k**, the HOMO and LUMO frontier molecular orbitals, along with derived descriptors such as the HOMO–LUMO gap, were calculated.

#### 4.4.2. Molecular Properties with Influence on Pharmacokinetics

To predict the pharmacokinetic behavior of the compounds, we employed SwissADME to evaluate their key molecular properties. This analysis included the calculation of TPSA, MlogP, and water solubility, as well as an assessment of compliance with Lipinski’s rule of five [25,57,58,59].

## 5. Conclusions

A series of thiazolyl-polyphenolic compounds was synthesized and subjected to complementary in silico and in vitro analyses to evaluate their antioxidant properties. In silico studies suggested that the non-planar conformation of these compounds minimizes the influence of the substituent in position 2 of the thiazole ring on the antioxidant activity of the catechol moiety in position 4. However, the presence of one or more phenolic hydroxyl groups in position 2 contributed to an overall enhancement of antioxidant activity. In vitro assays confirmed these findings, with compounds **7j** and **7k** exhibiting significantly lower IC_50_ values than the reference antioxidants, demonstrating high efficacy in both radical scavenging and electron transfer mechanisms. These results highlight the potential of thiazolyl-polyphenolic compounds for further development as therapeutic agents. Future research could focus on the structural modifications in position 2 of the thiazole ring to optimize other physicochemical and biological properties.

A comparative analysis of antioxidant activity, assessed through both in vitro assays and in silico calculations, reveals a complex relationship between predicted and observed behaviors. While the in silico methods offer valuable insights into potential activity, a single descriptor cannot describe perfectly the whole series of compounds, to correlate it with the in vitro results. Specifically, in silico HOMO–LUMO analysis indicated compound **7a** as the most potent antioxidant, exhibiting the smallest energy gap in two of the three simulated environments, whereas compound **7f** was predicted to be the least active, from a point of view of electron transfer. Conversely, in silico BDEs calculations suggested compound **7d** as the most promising antioxidant, displaying the lowest BDE values across all three environments, from an antiradical point of view. However, in vitro assays, confirming antioxidant activity, did not correlate perfectly with the ranking predicted by of a single in silico study. This can be attributed to two possible factors: one, the antioxidant activity of a compound can be the result of combined mechanisms-electron transfer and hydrogen transfer and, second, the BDE calculations considered only the first step of radicalization of the studied antioxidants. Because some of the present compounds have multiple OH phenol groups, such as **7d**–**f** and **7j**–**k**, found on different phenol rings, at a relative high distance between them, they could radicalize in multiple steps, possibility which was not taken into account by the present study, due to the complexity of the possible combinations of radicals in the second step of radicalization.

## Figures and Tables

**Table 1:** Description of the R_1_–R_5_ fragments.

Compound	R_1_	R_2_	R_3_	R_4_	R_5_
**7a**	OH	H	H	H	H
**7b**	H	OH	H	H	H
**7c**	H	H	OH	H	H
**7d**	H	OH	OH	H	H
**7e**	OH	H	OH	H	H
7f *	OH	H	OH	H	H
**7g**	H	OH	-OCH_3_	H	H
**7h**	H	-OCH_3_	OH	H	H
**7i**	H	-OCH_2_CH_3_	OH	H	H
**7j**	OH	OH	OH	H	H
**7k**	OH	H	OH	H	OH

* For compound **7f**, R= -CH_3_

**Table 2:** Half-maximal inhibitory concentrations (IC_50_) of compounds **7a**–**k**, against the DPPH^•^ and ABTS^•+^ (µM).

Compound	DPPH^•^	ABTS^•+^
**7a**	53.98	11.54
**7b**	50.94	7.90
**7c**	39.90	9.63
**7d**	30.49	5.98
**7e**	35.84	2.70
**7f**	42.14	3.22
**7g**	36.85	4.54
**7h**	39.24	10.19
**7i**	37.78	7.83
**7j**	25.29	2.86
**7k**	35.03	2.50
**Ascorbic acid**	53.49	NT
**Trolox**	38.01	15.87

NT = not tested; DPPH^•^ = 2,2-diphenyl-1-picrylhydrazyl; ABTS^•+^ = 2,2′-azinobis-3-ethylbenzothiazoline-6-sulfonic acid.

**Table 3:** Electron transfer assay results for compounds **7a**–**k** expressed as ascorbic acid/Trolox equivalents.

Compound	TAC	RP		FRAP	CUPRAC
	Eq Ascorbic Acid	Eq Ascorbic Acid	Eq Trolox	Eq Trolox	Eq Trolox
**7a**	1.03	3.28	2.72	1.42	3.32
**7b**	1.05	3.67	3.05	1.48	5.26
**7c**	1.02	3.25	2.70	1.41	5.46
**7d**	1.41	3.47	2.88	1.32	3.79
**7e**	1.27	3.52	2.92	1.56	5.79
**7f**	1.58	3.32	2.76	1.49	3.97
**7g**	1.03	3.71	3.08	1.47	3.74
**7h**	1.06	3.21	2.67	1.32	4.60
**7i**	1.05	3.82	3.18	1.37	5.56
**7j**	2.38	4.24	3.53	1.66	5.87
**7k**	1.78	3.84	3.19	1.59	6.03

TAC = Total Antioxidant Capacity; RP = Reducing Power; FRAP = Ferric-Reducing Antioxidant Potential; CUPRAC = CUPric-Reducing Antioxidant Capacity.

**Table 4:** The energy level of the frontier molecular orbitals HOMO, LUMO, and the HOMO–LUMO gap in vacuum, non-polar environment and water for compounds **7a**–**k** (eV).

Compound	Vacuum			Non-Polar			Water		
	HOMO	LUMO	Gap	HOMO	LUMO	Gap	HOMO	LUMO	Gap
**7a**	−5.23	−1.52	3.71	−5.24	−1.59	3.65	−5.25	−1.61	3.64
**7b**	−5.05	−1.36	3.69	−5.18	−1.52	3.66	−5.21	−1.56	3.65
**7c**	−4.93	−1.20	3.73	−5.06	−1.36	3.70	−5.09	−1.39	3.70
**7d**	−4.93	−1.19	3.74	−5.06	−1.36	3.70	−5.09	−1.40	3.69
**7e**	−5.06	−1.30	3.76	−5.14	−1.42	3.72	−5.15	−1.44	3.71
**7f**	−5.10	−1.28	3.82	−5.13	−1.33	3.80	−5.14	−1.34	3.80
**7g**	−4.87	−1.15	3.72	−5.04	−1.36	3.68	−5.08	−1.40	3.68
**7h**	−4.86	−1.17	3.69	−5.03	−1.36	3.67	−5.06	−1.40	3.66
**7i**	−4.85	−1.15	3.70	−5.02	−1.35	3.67	−5.06	−1.40	3.66
**7j**	−5.17	−1.43	3.74	−5.17	−1.46	3.71	−5.16	−1.46	3.70
**7k**	−5.11	−1.40	3.71	−5.15	−1.46	3.69	−5.15	−1.47	3.68

**Table 5:** The BDE for the OH phenol groups from compounds **7a**–**k** in vacuum (kcal/mol).

Compound	O-H_1_	O-H_2_	O-H_3_	O-H_4_	O-H_5_	O-H_6_ *
**7a**	69.23	71.55	82.99	-	-	-
**7b**	68.41	71.23	-	79.29	-	-
**7c**	67.34	70.98	-	-	73.08	-
**7d**	68.01	70.99	-	68.67	64.58	-
**7e**	68.31	70.82	87.18	-	75.84	-
**7f**	68.80	71.73	84.29	-	77.02	-
**7g**	67.80	70.83	-	76.16	-	-
**7h**	68.37	70.83	-	-	73.27	-
**7i**	68.28	70.73	-	-	73.02	-
**7j**	68.47	71.27	86.13	71.69	77.22	-
**7k**	68.54	71.33	78.87	-	73.36	72.08

* Considered only when O-H_3_ is present, - not substituted in the respective site with OH group.

**Table 6:** The BDE for the OH phenol groups from compounds **7a**–**k** in nonpolar (ε = 7.43) environment (kcal/mol).

Compound	O-H_1_	O-H_2_	O-H_3_	O-H_4_	O-H_5_	O-H_6_ *
**7a**	69.38	71.92	78.74	-	-	-
**7b**	69.31	72.12	-	79.21	-	-
**7c**	68.91	71.87	-	-	71.64	-
**7d**	68.93	71.91	-	69.95	65.31	-
**7e**	69.16	71.80	88.68	-	73.25	-
**7f**	69.01	71.99	80.63	-	74.19	-
**7g**	68.89	71.89	-	76.07	-	-
**7h**	69.21	71.74	-	-	72.46	-
**7i**	69.17	71.68	-	-	72.29	-
**7j**	69.04	72.02	83.34	69.97	72.71	-
**7k**	69.30	71.93	82.18	-	70.75	70.44

* Considered only when O-H_3_ is present, - not substituted in the respective site with OH group.

**Table 7:** The BDE for the OH phenol groups from compounds **7a**–**k** in water (kcal/mol).

Compound	O-H_1_	O-H_2_	O-H_3_	O-H_4_	O-H_5_	O-H_6_ *
**7a**	71.74	74.31	80.09	-	-	-
**7b**	71.79	74.59	-	81.45	-	-
**7c**	71.40	74.38	-	-	73.49	-
**7d**	71.43	74.42	-	72.48	67.68	-
**7e**	71.68	74.28	91.27	-	74.84	-
**7f**	71.35	74.33	82.11	-	75.65	-
**7g**	71.42	74.42	-	78.51	-	-
**7h**	71.69	74.24	-	-	74.55	-
**7i**	71.62	74.16	-	-	74.34	-
**7j**	71.30	74.33	84.92	71.86	73.74	-
**7k**	71.70	74.29	84.97	-	72.23	72.08

* Considered only when O-H_3_ is present, - not substituted in the respective site with OH group.

**Table 8:** Molecular and pharmacokinetic properties of compounds **7a**–**k**: molecular weight (MW), number of hydrogen bond acceptors (HBA), number of hydrogen bond donors (HBD), topological polar surface area (TPSA), logP calculated by the Moriguchi method (MLogP), water solubility, and Lipinski violations.

Compound	MW	Rotatable Bonds	HBA	HBD	TPSA (Å^2^)	MLogP	Water Solubility (µg/mL)	Lipinski Violations
**7a**	341.38	3	5	3	118.58	1.48	2.54	0
**7b**	341.38	3	5	3	118.58	1.48	2.54	0
**7c**	341.38	3	5	3	118.58	1.48	2.54	0
**7d**	357.38	3	6	4	138.81	0.95	3.74	0
**7e**	357.38	3	6	4	138.81	0.95	3.74	0
**7f**	371.41	3	6	4	138.81	1.19	2.42	0
**7g**	371.41	4	6	3	127.81	1.19	2.40	0
**7h**	371.41	4	6	3	127.81	1.19	2.40	0
**7i**	385.44	5	6	3	127.81	1.42	1.47	0
**7j**	373.38	3	7	5	159.04	0.43	5.40	0
**7k**	373.38	3	7	5	159.04	0.43	5.40	0

## Data Availability

Data is contained within the article or Supplementary Materials.

## Supplementary Materials

The following supporting information can be downloaded at: https://www.mdpi.com/article/10.3390/molecules30061345/s1, Figures S1–S11: The IR spectra for compounds **7a**–**k**; Figures S12–S22: The 1H-NMR spectrum for compounds **7a**–**k**; Figures S23–S33: The 13C-NMR spectrum for compounds **7a**–**k**; Figures S34–S44: The MS spectrum for compounds **7a**–**k**; Table S1: The depiction of HOMO and LUMO for the compounds **7a**–**k**; Table S2: The electrostatic potential map for compounds **7a**–**k**.
