# Supplementary material for: Synthesis, Characterization, and Antioxidant Activity Evaluation of New N-Methyl Substituted Thiazole-Derived Polyphenolic Compounds
Source: Molecules. 2025 Mar 17;30(6):1345. doi: 10.3390/molecules30061345 (PMC11944991; doi:10.3390/molecules30061345)
Supplement: Supplementary file 1 [file molecules-30-01345-s001.zip › molecules-3503068-supplementary.pdf]

molecules

MDPI

Synthesis, Characterization, and Antioxidant Activity Evaluation of New N-Methyl Substituted Thiazole-Derived Polyphenolic Compounds

Alexandra Cătălina Cornea [1], Gabriel Marc [2,*], Ioana Ionuț [1], Cristina Moldovan [1], Anca Stana [1], Smaranda Dafina Oniga [3], Adrian Pîrnău [4], Laurian Vlase [5], Ilioara Oniga [6] and Ovidiu Oniga [1]

1. Figures

1.1. The IR spectra

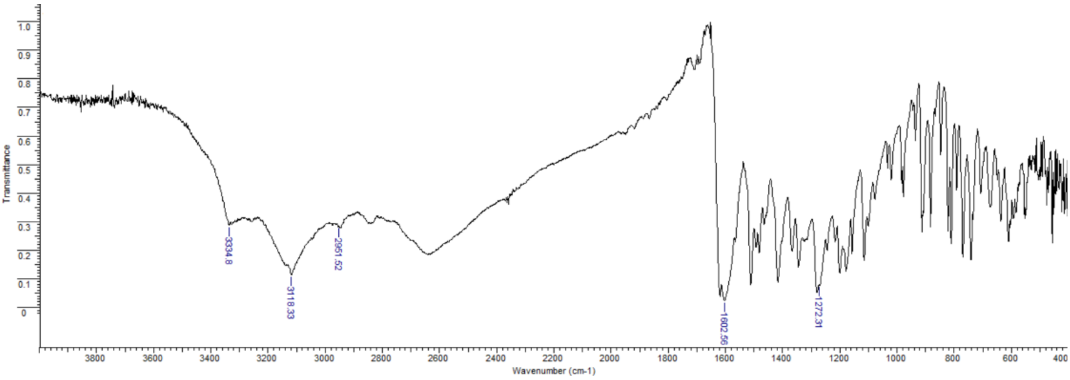

Figure S1. The IR spectrum for the compound **7a**

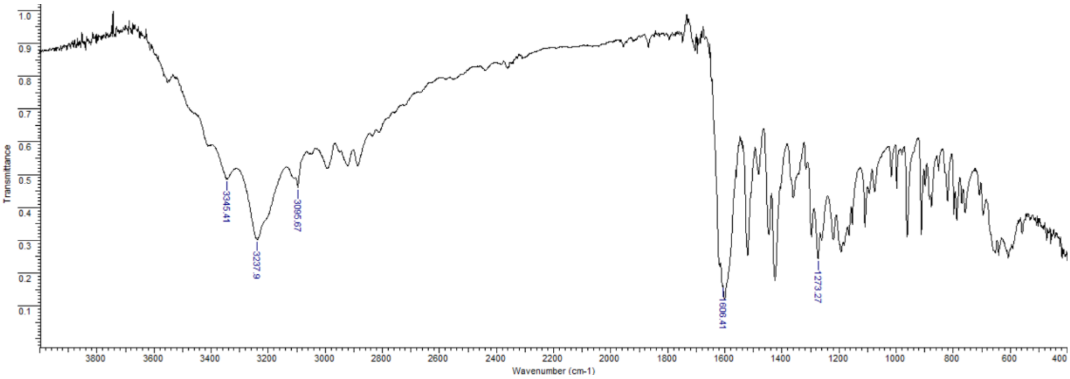

Figure S2. The IR spectrum for the compound **7b**

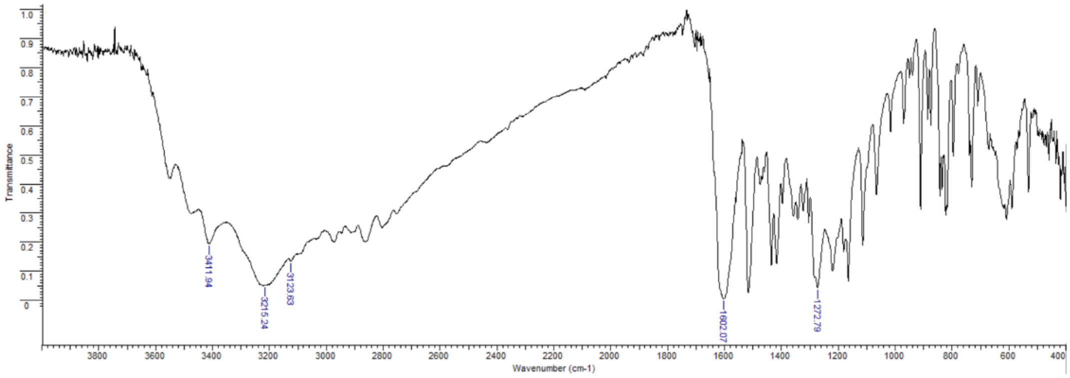

Figure S3. The IR spectrum for the compound **7c**

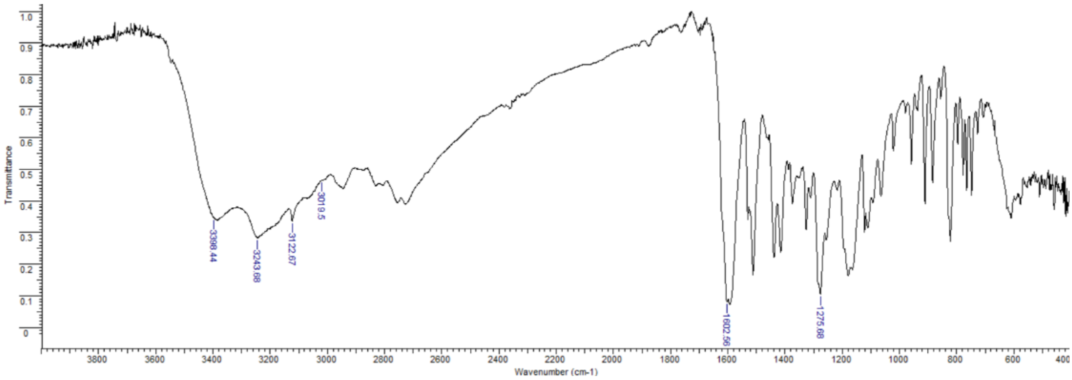

Figure S4. The IR spectrum for the compound **7d**

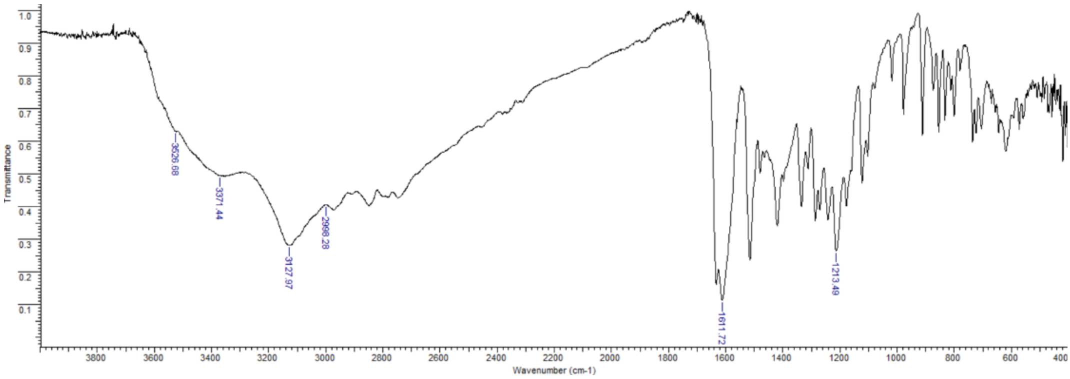

Figure S5. The IR spectrum for the compound **7e**

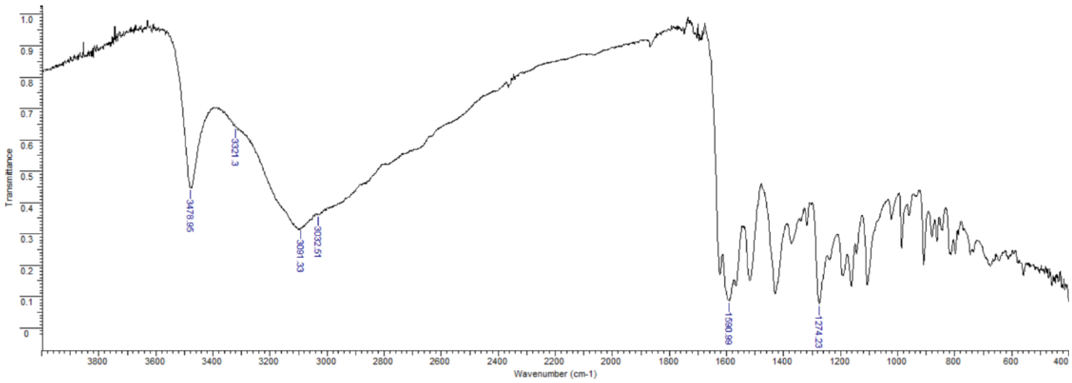

Figure S6. The IR spectrum for the compound **7f**

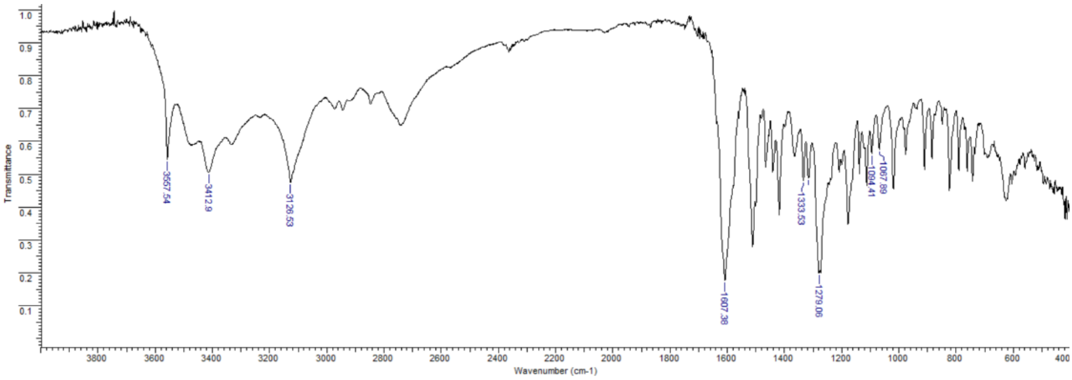

Figure S7. The IR spectrum for the compound **7g**

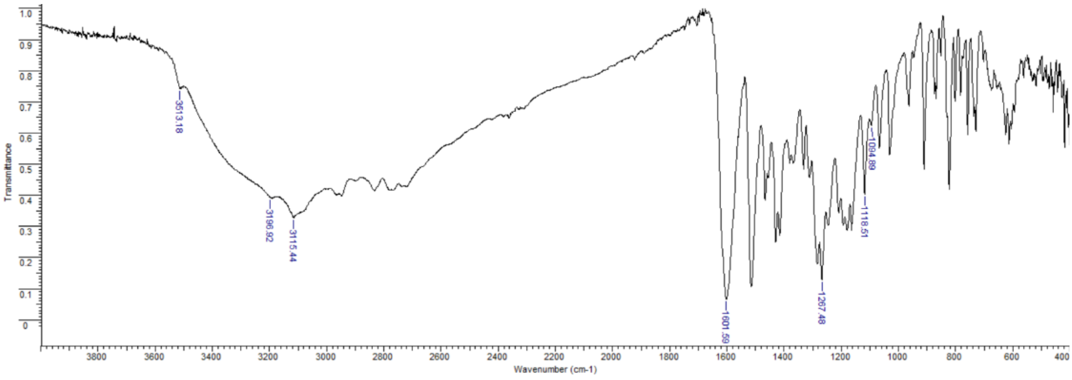

Figure S8. The IR spectrum for the compound **7h**

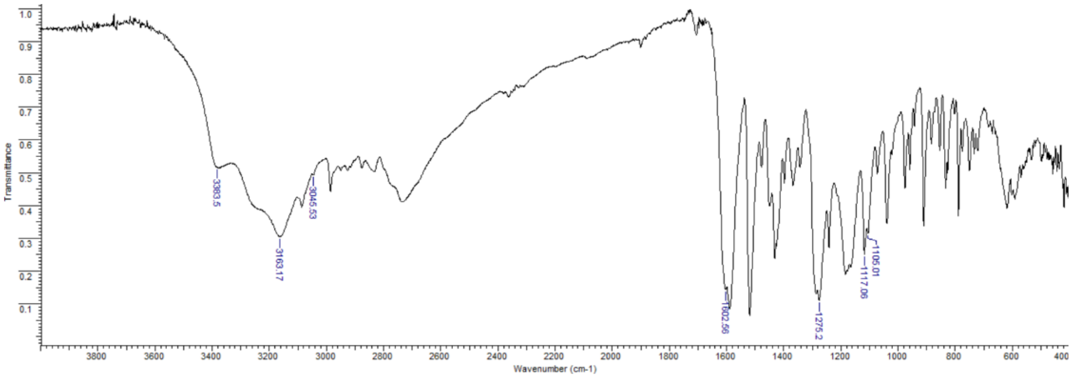

Figure S9. The IR spectrum for the compound **7i**

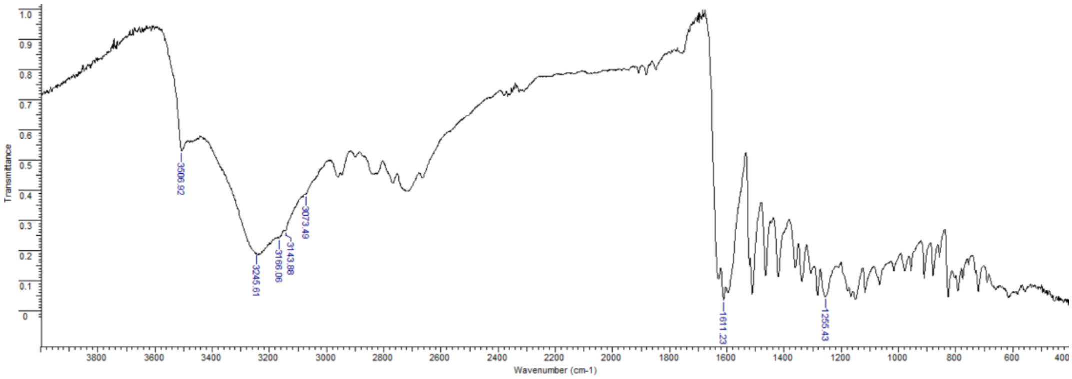

Figure S10. The IR spectrum for the compound **7j**

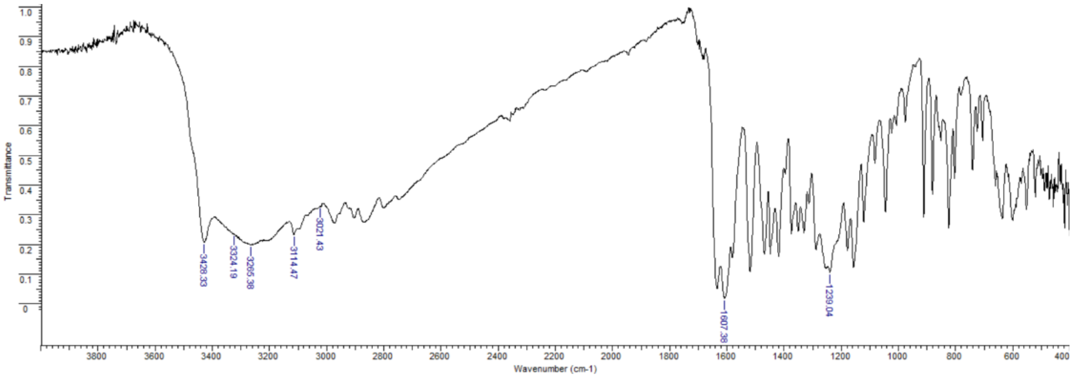

Figure S11. The IR spectrum for the compound **7k**

1.2 The ¹H-NMR spectra

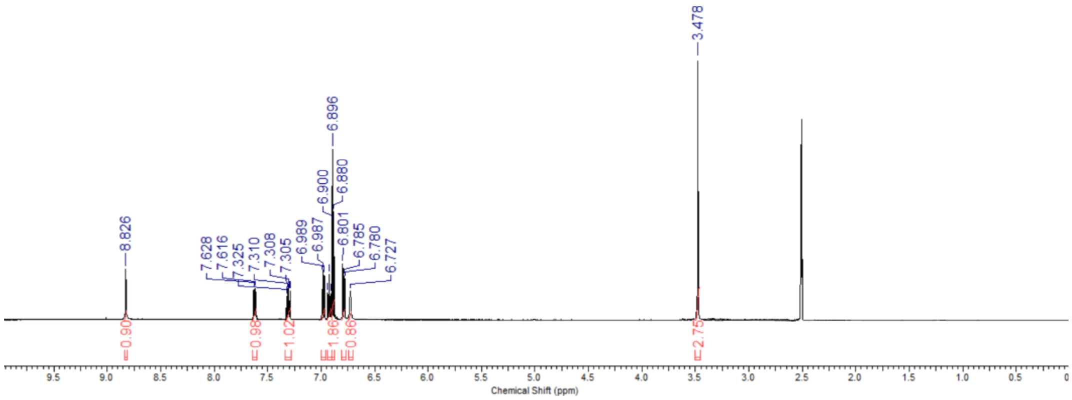

Figure S12. The 1H-NMR spectrum for the compound **7a**

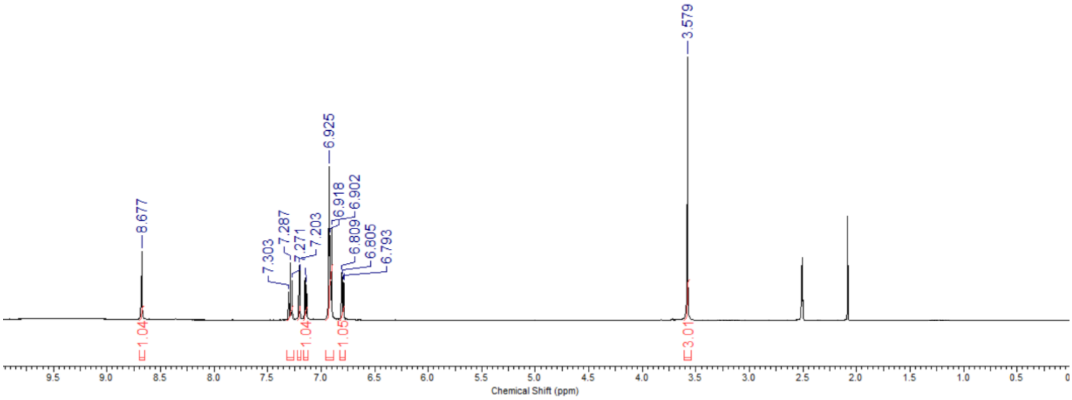

Figure S13. The 1H-NMR spectrum for the compound **7b**

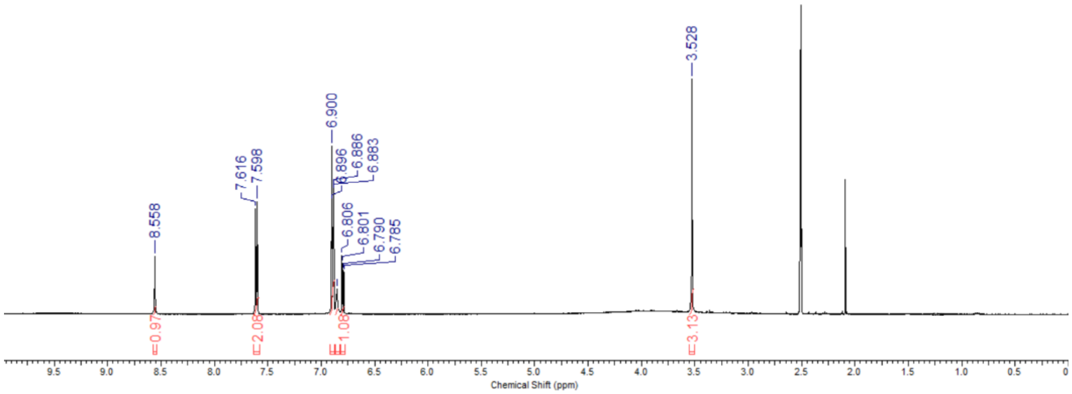

Figure S14. The 1H-NMR spectrum for the compound **7c**

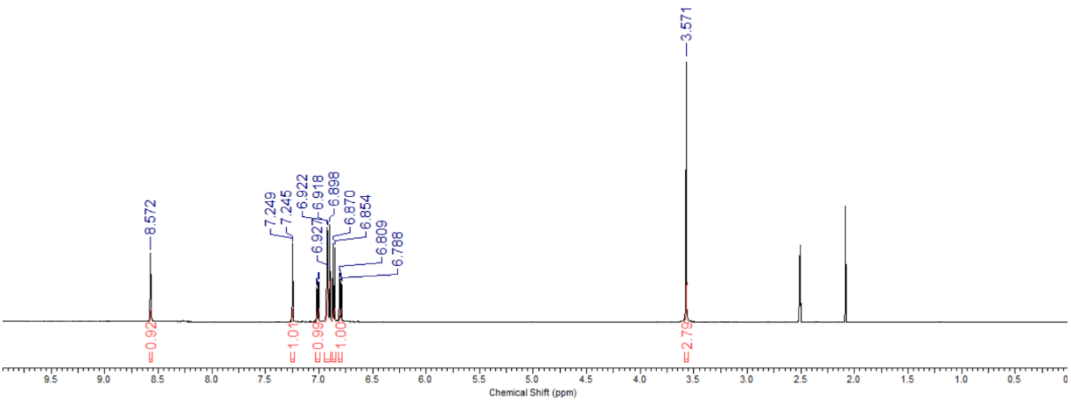

Figure S15. The 1H-NMR spectrum for the compound **7d**

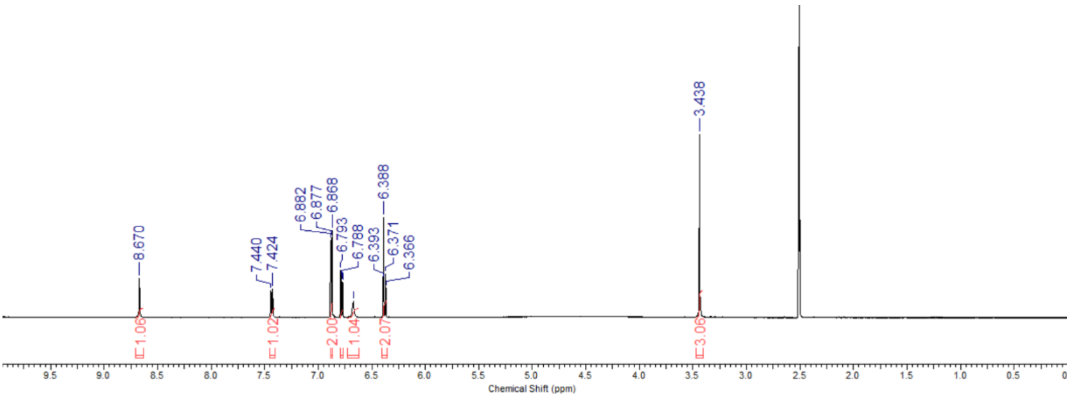

Figure S16. The 1H-NMR spectrum for the compound **7e**

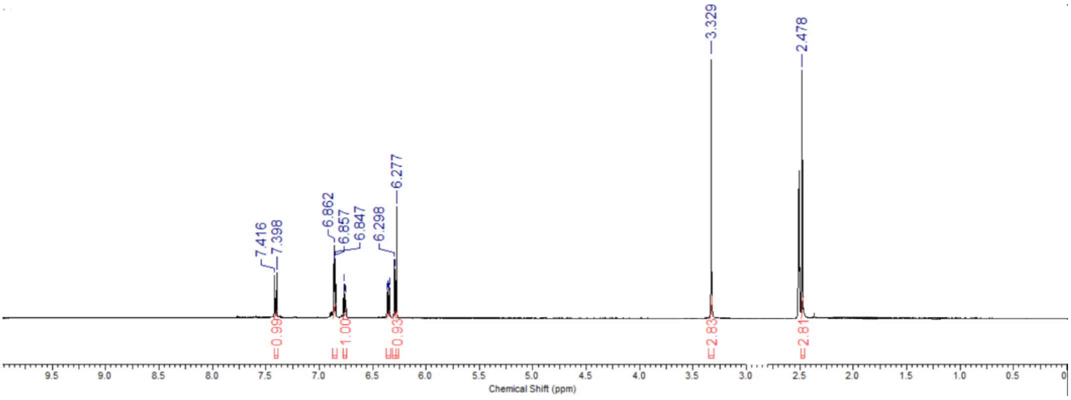

Figure S17. The 1H-NMR spectrum for the compound **7f**

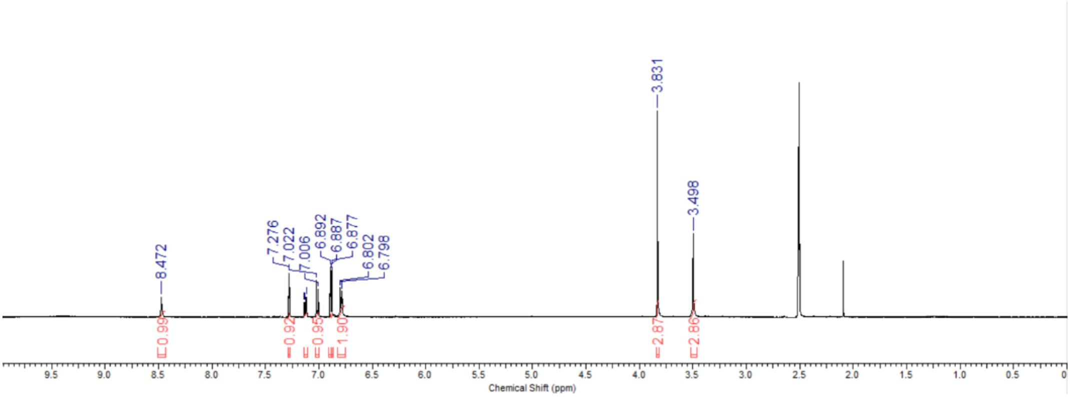

Figure S18. The 1H-NMR spectrum for the compound **7g**

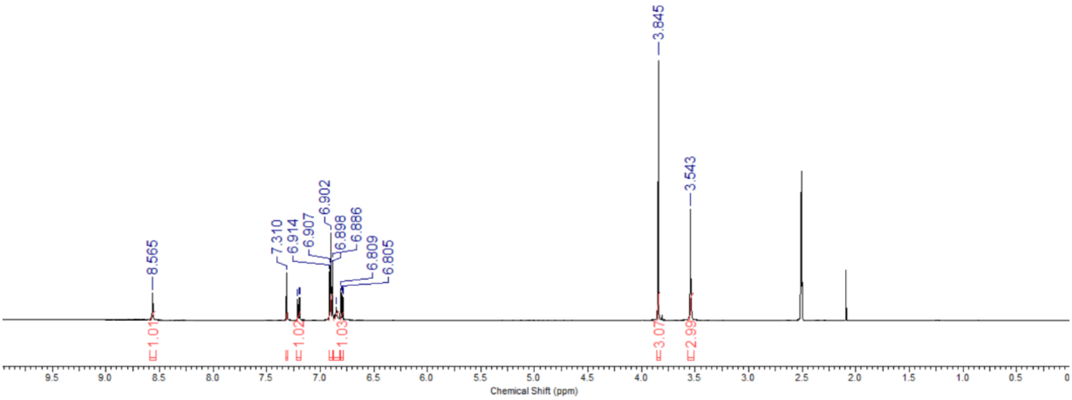

Figure S19. The 1H-NMR spectrum for the compound **7h**

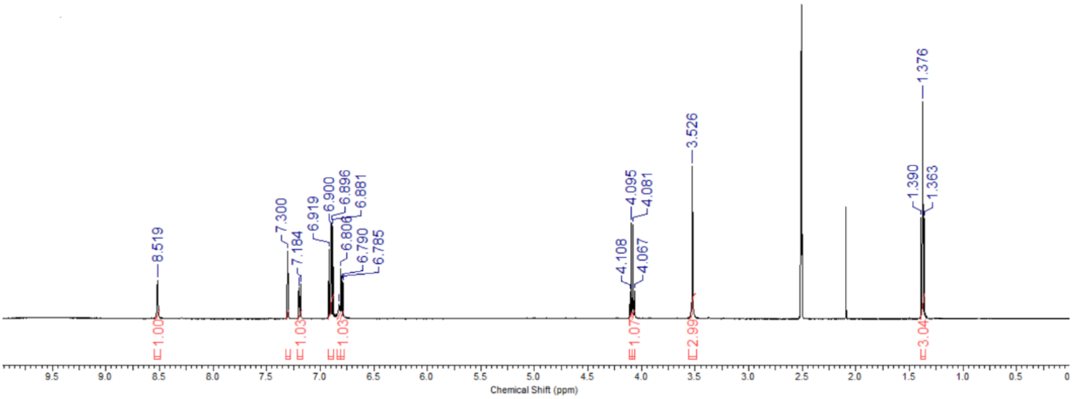

Figure S20. The 1H-NMR spectrum for the compound **7i**

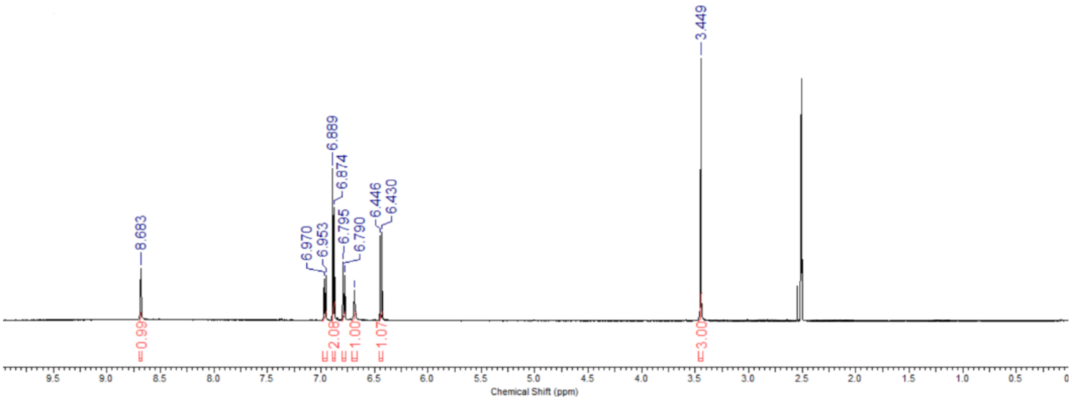

Figure S21. The 1H-NMR spectrum for the compound **7j**

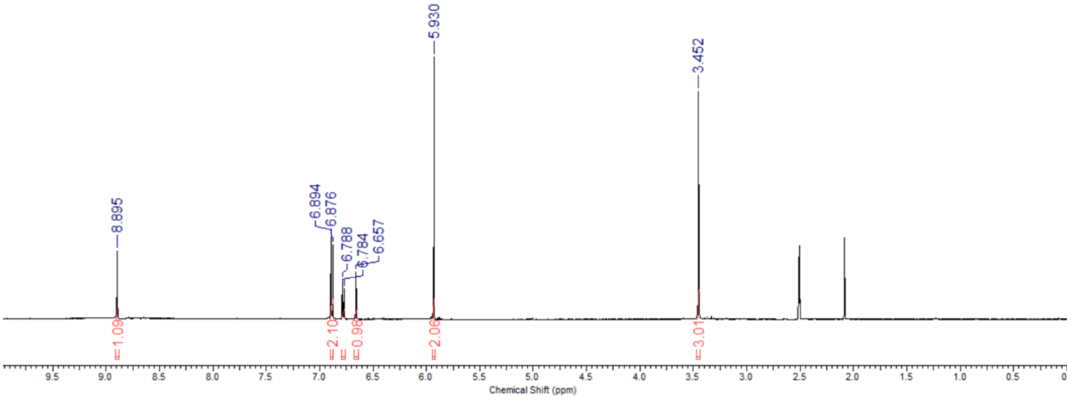

Figure S22. The 1H-NMR spectrum for the compound **7k**

1.3 The ^{13}C-NMR spectra

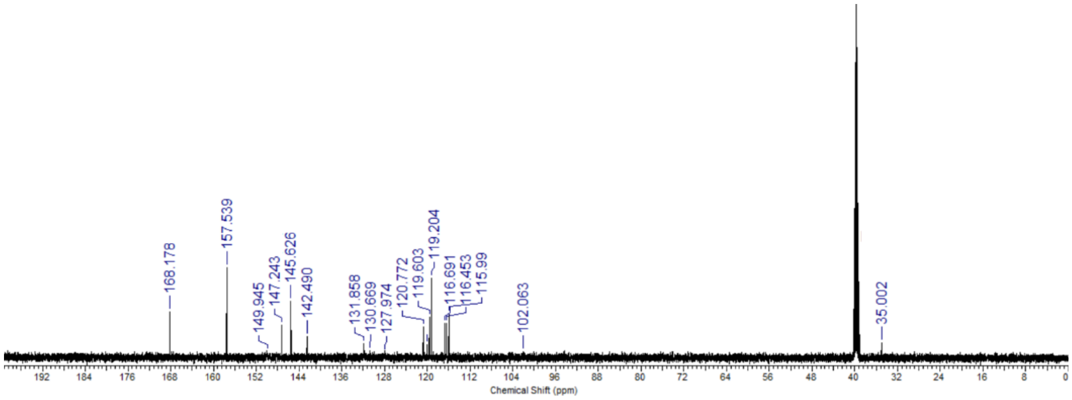

Figure S23. The ^{13}C-NMR spectrum for the compound **7a**

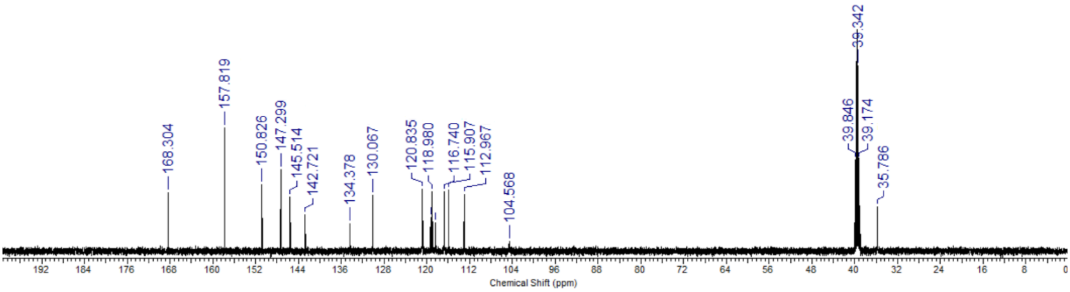

Figure S24. The ^{13}C-NMR spectrum for the compound **7b**

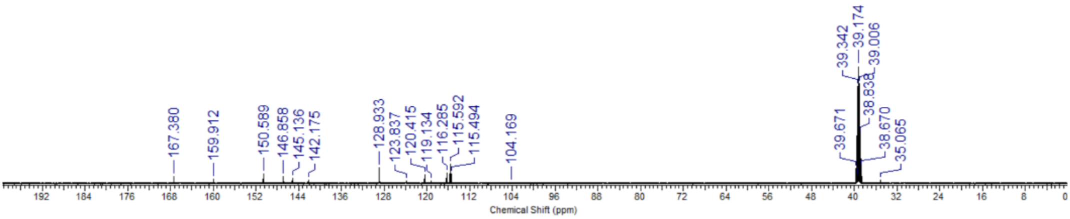

Figure S25. The ^{13}C-NMR spectrum for the compound **7c**

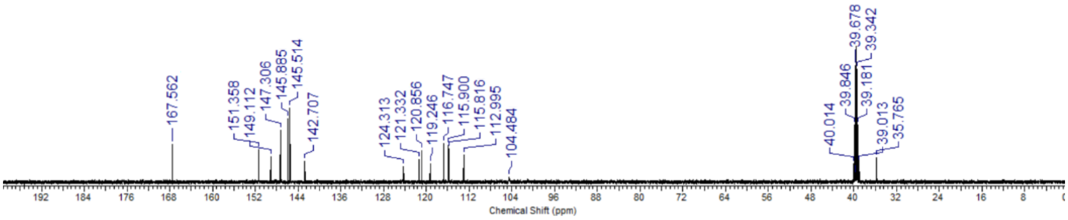

Figure S26. The ^{13}C-NMR spectrum for the compound **7d**

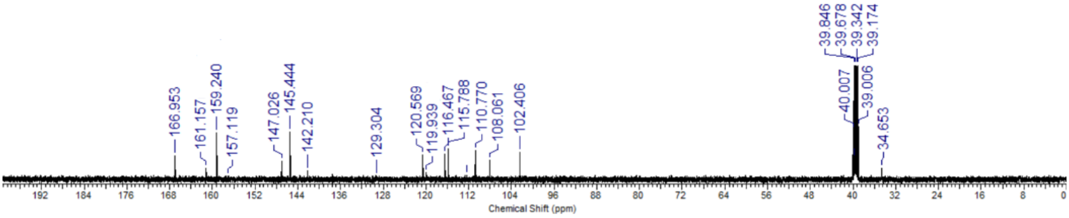

Figure S27. The ^{13}C-NMR spectrum for the compound **7e**

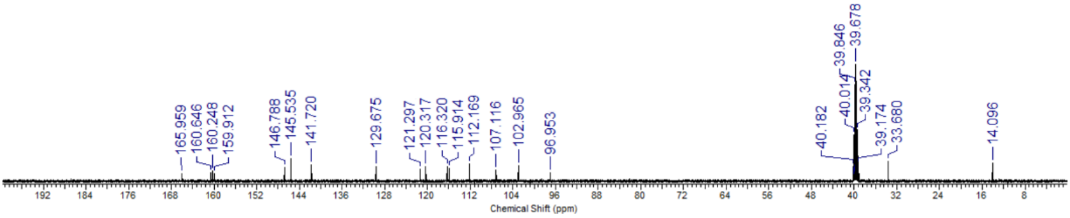

Figure S28. The ^{13}C-NMR spectrum for the compound **7f**

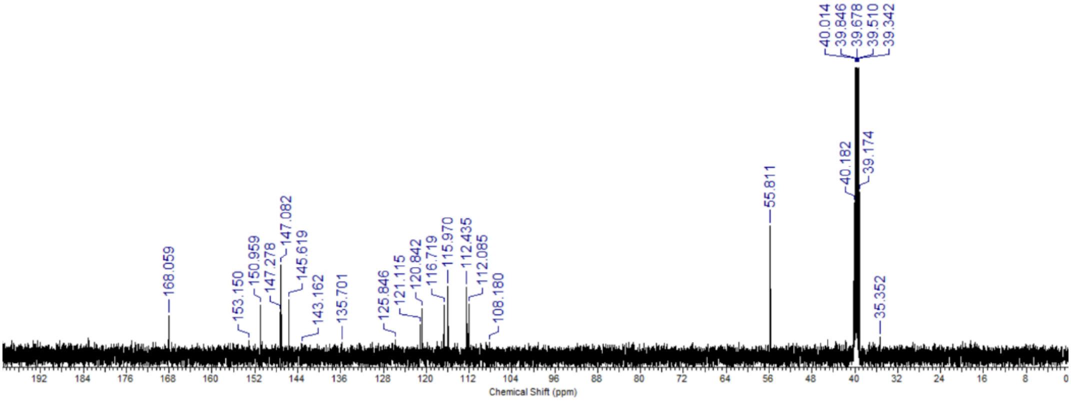

Figure S29. The ^{13}C-NMR spectrum for the compound **7g**

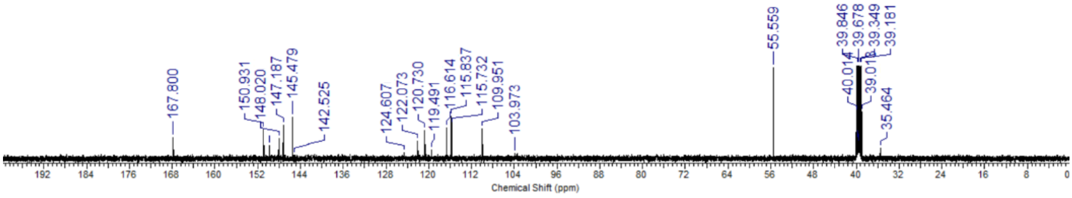

Figure S30. The ^{13}C-NMR spectrum for the compound **7h**

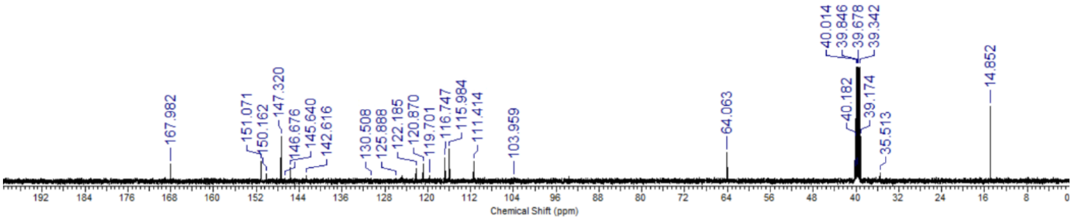

Figure S31. The ^{13}C-NMR spectrum for the compound **7i**

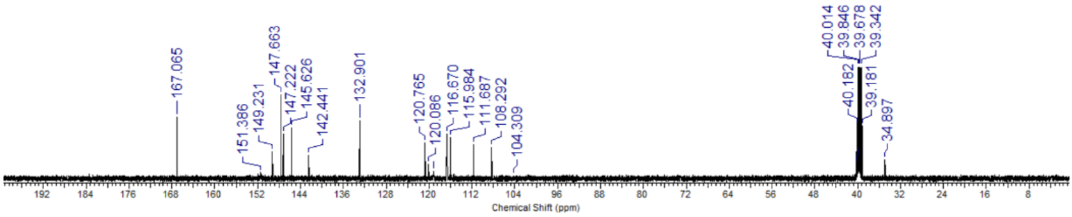

Figure S32. The ^{13}C-NMR spectrum for the compound **7j**

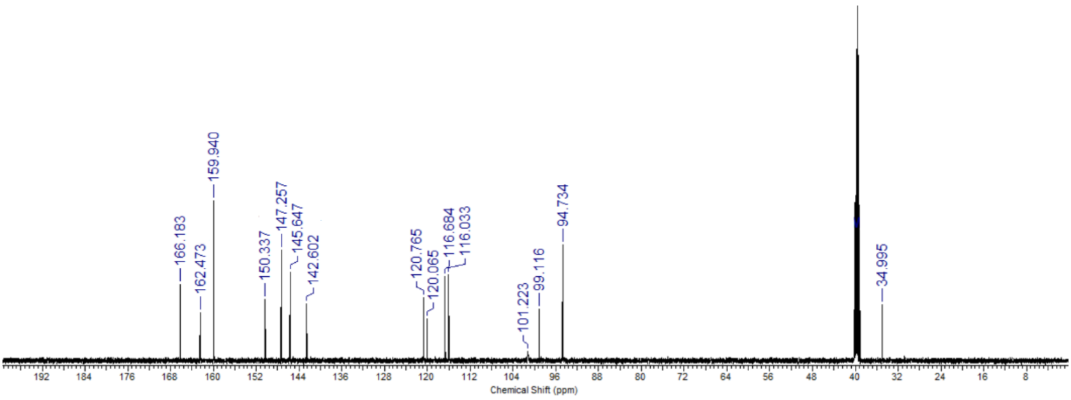

Figure S33. The ^{13}C-NMR spectrum for the compound **7k**

1.4 The MS spectra

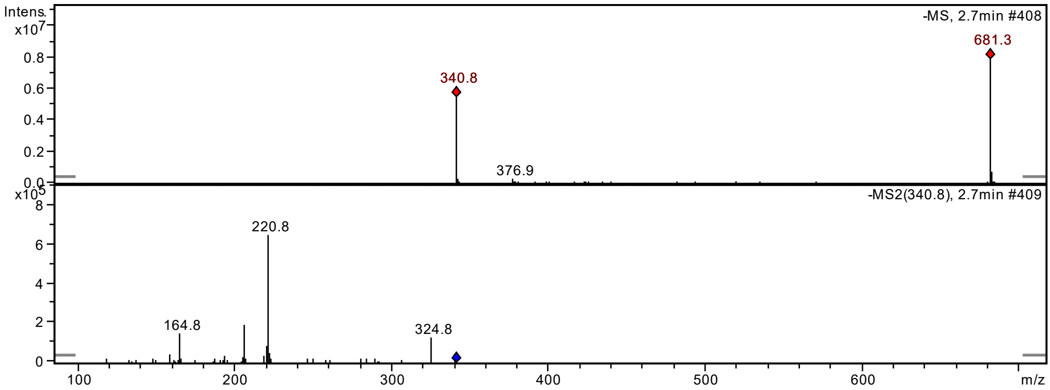

Figure S34. The MS spectrum for the compound **7a**

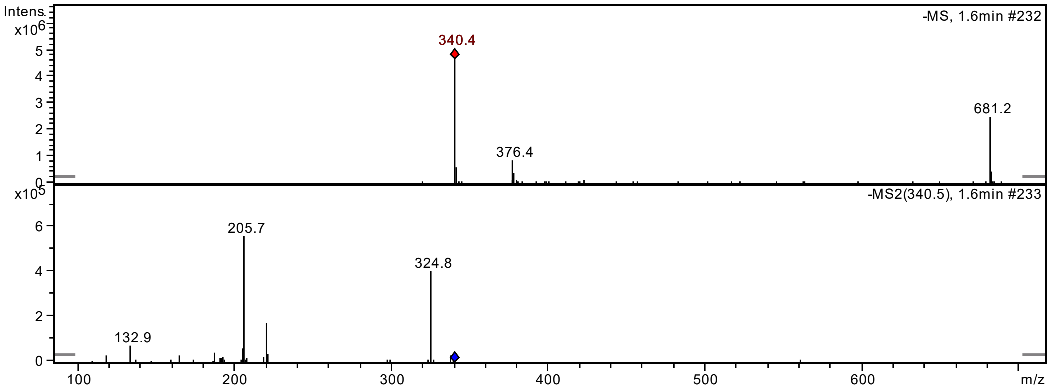

Figure S35. The MS spectrum for the compound **7b**

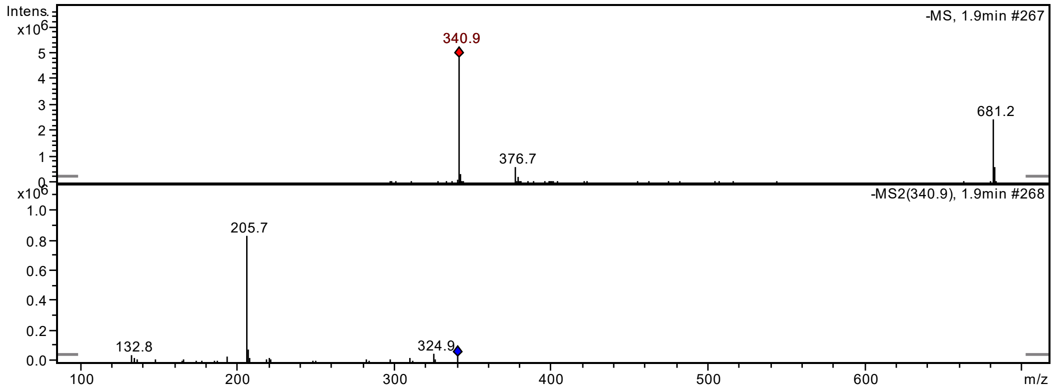

Figure S36. The MS spectrum for the compound **7c**

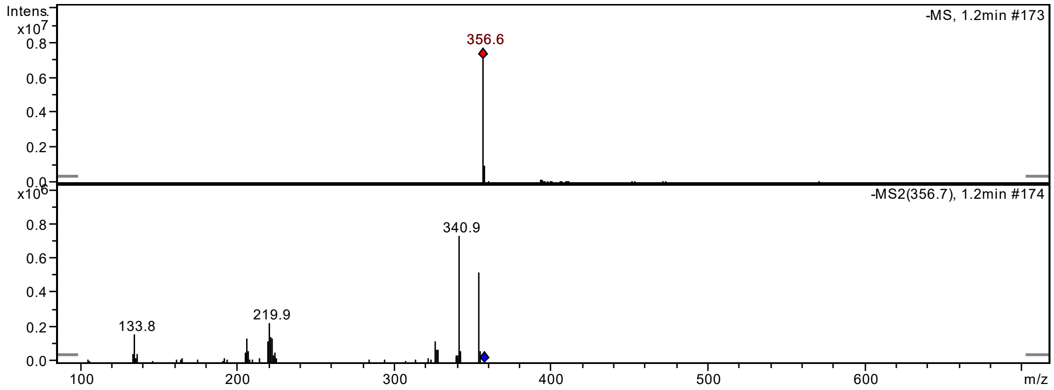

Figure S37. The MS spectrum for the compound **7d**

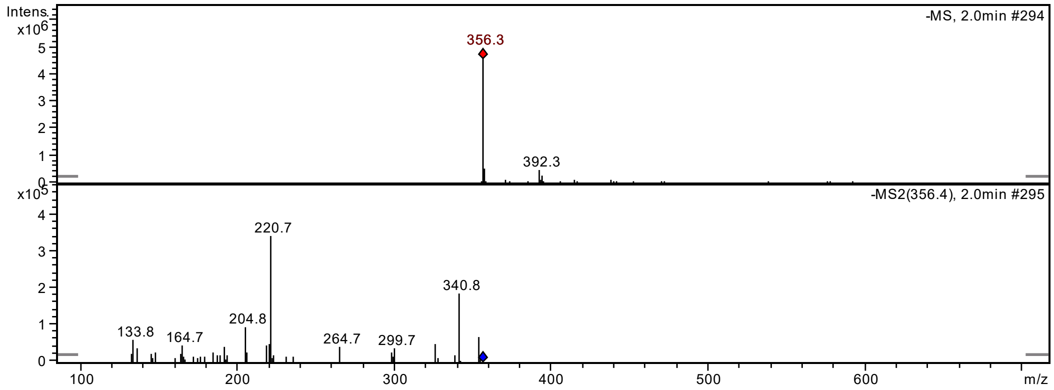

Figure S38. The MS spectrum for the compound **7e**

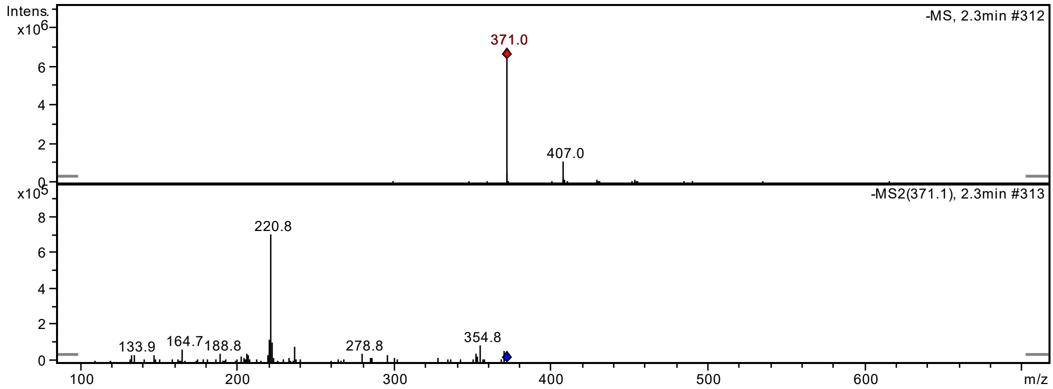

Figure S39. The MS spectrum for the compound **7f**

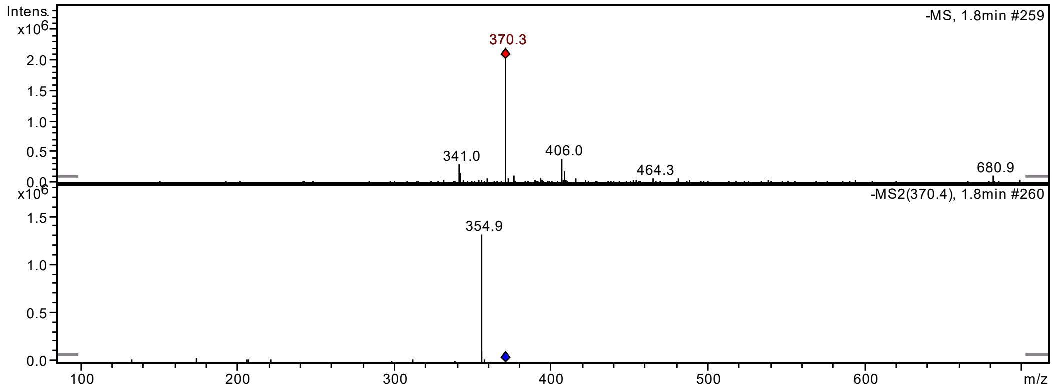

Figure S40. The MS spectrum for the compound **7g**

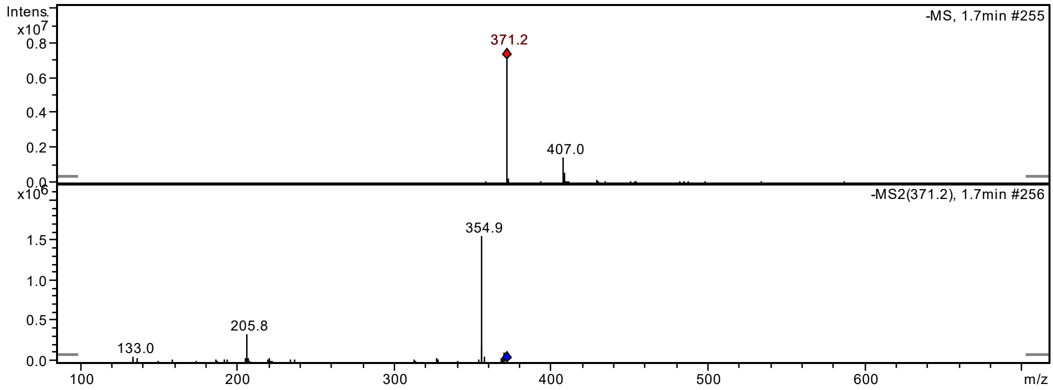

Figure S41. The MS spectrum for the compound **7h**

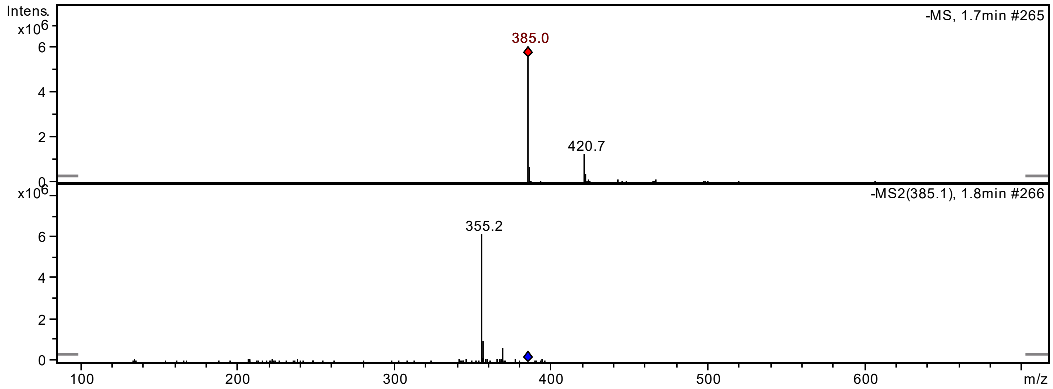

Figure S42. The MS spectrum for the compound **7i**

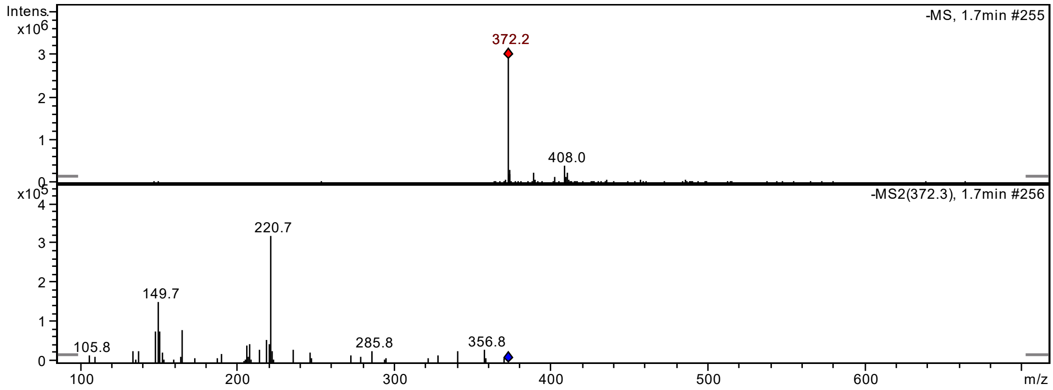

Figure S43. The MS spectrum for the compound **7j**

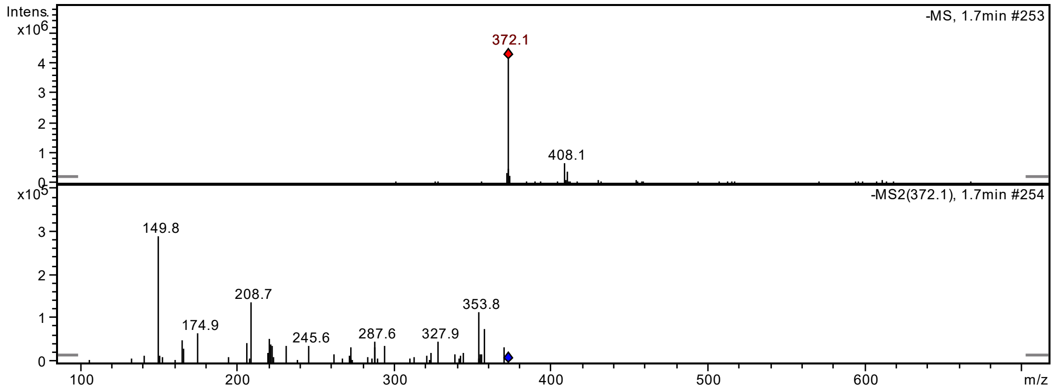

Figure S44. The MS spectrum for the compound **7k**

2. Tables

*2.1. The depiction of HOMO and LUMO and the depiction of the spin density maps of compounds **7a-k***

Table S1. The depiction of HOMO and LUMO for the compounds **7a-k**

Compound	HOMO	LUMO
**7a**	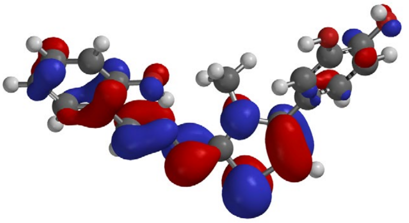	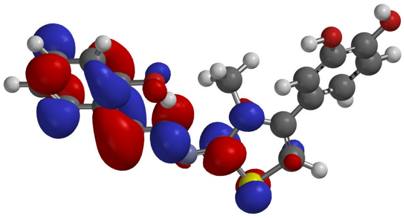
**7b**	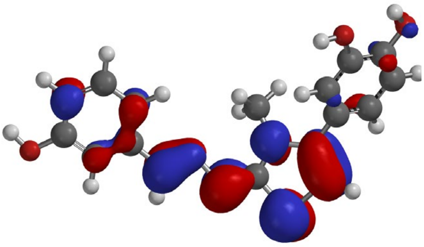	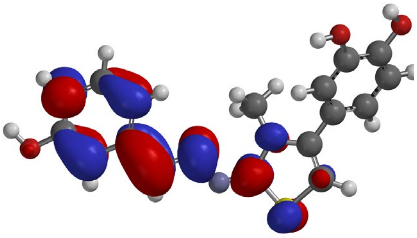
**7c**	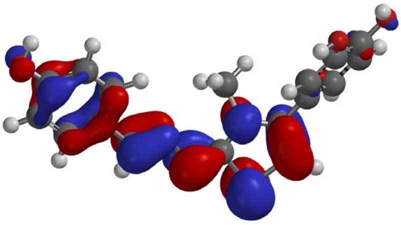	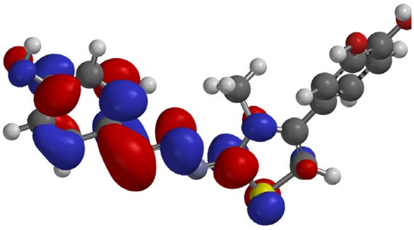
**7d**	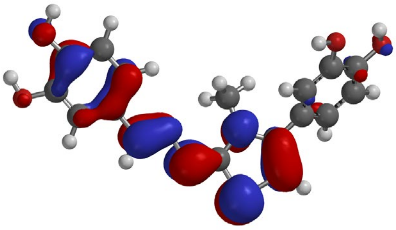	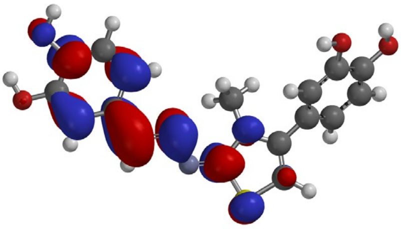

**7e**	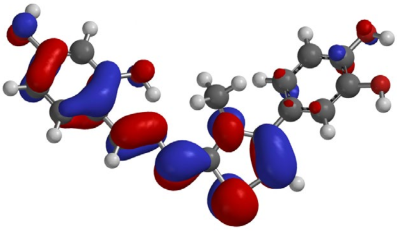	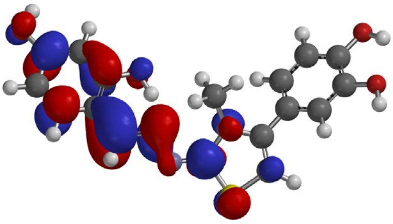
**7f**	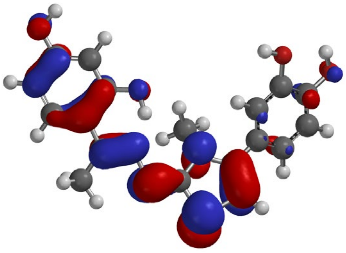	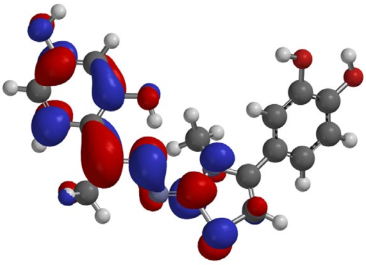
**7g**	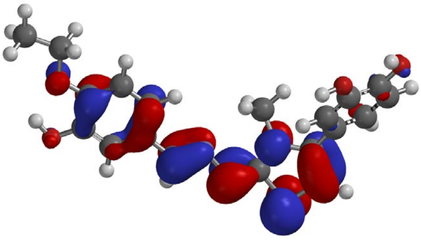	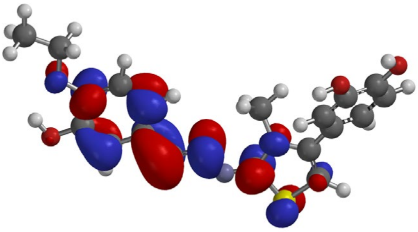
**7h**	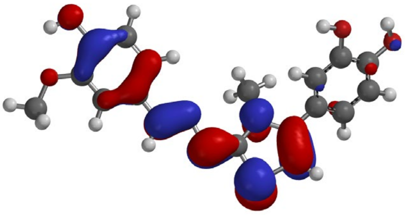	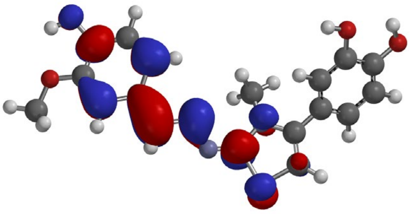
**7i**	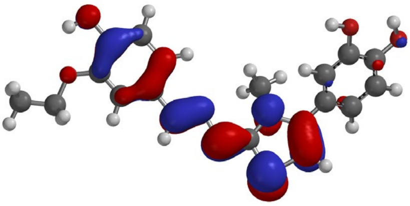	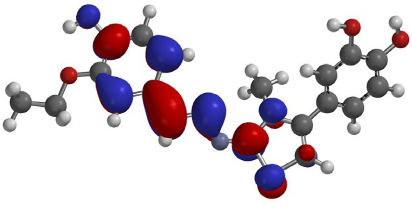

7j	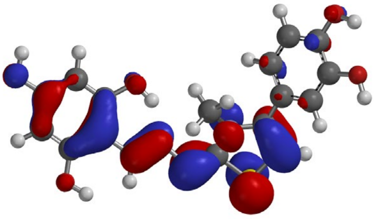	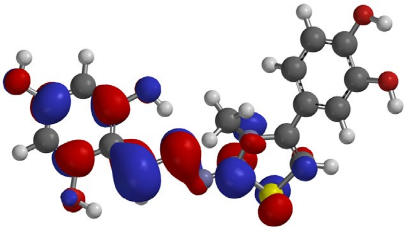
7k	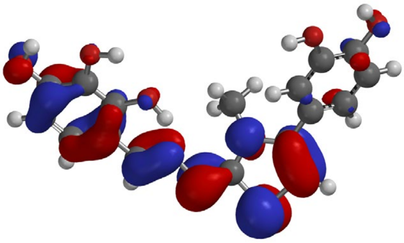	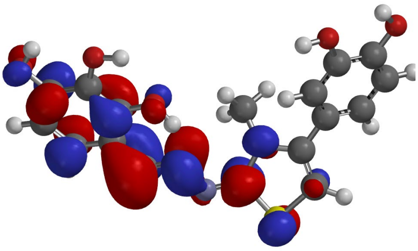

Table S2: The electrostatic potential map for compounds **7a-k**.

Compound	EPM
7a	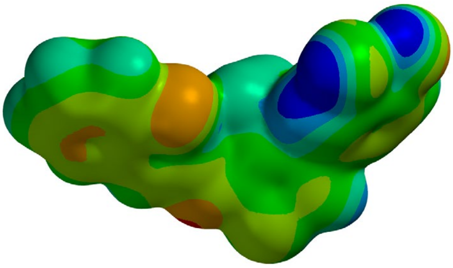
7b	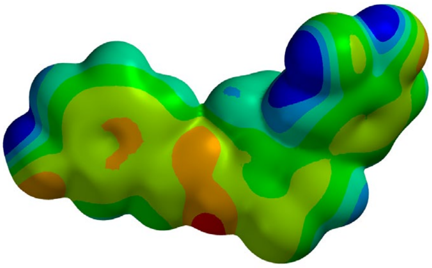

**7c**	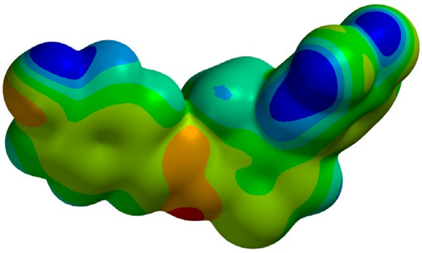
**7d**	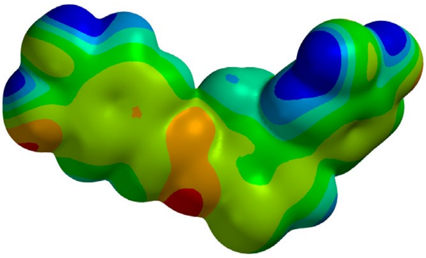
**7e**	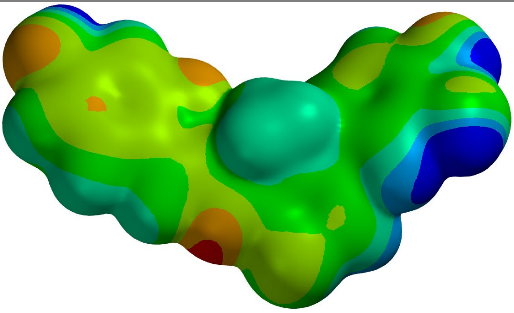
**7f**	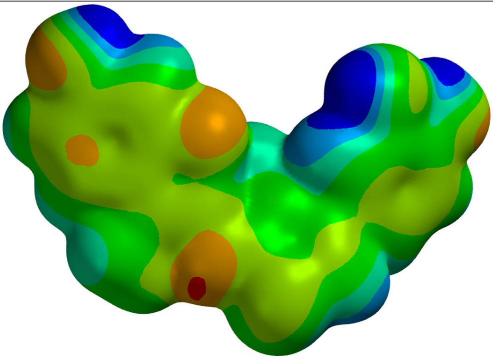

**7g**	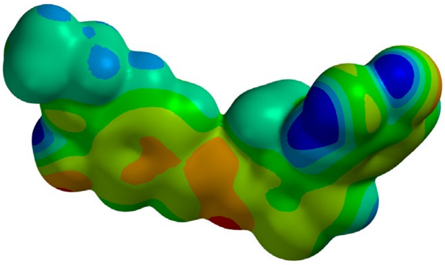
**7h**	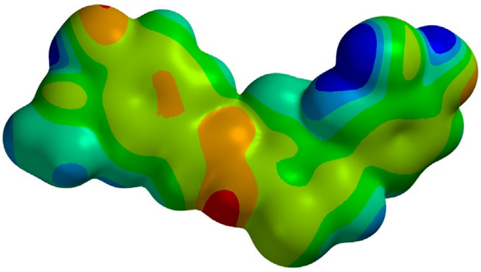
**7i**	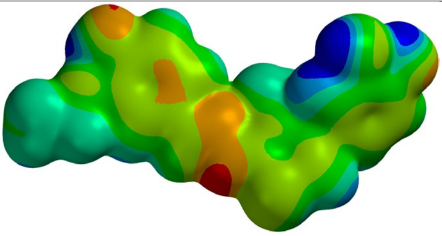
**7j**	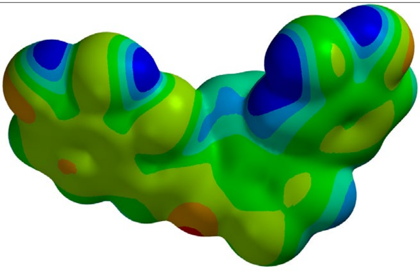
**7k**	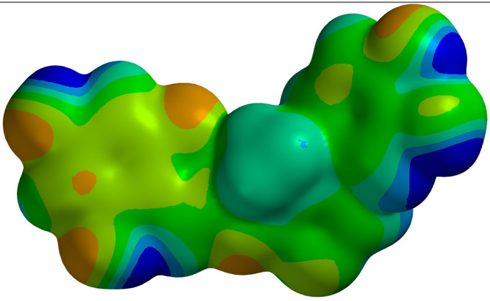
